# BDKRB2 is a novel EMT-related biomarker and predicts poor survival in glioma

**DOI:** 10.18632/aging.202614

**Published:** 2021-03-03

**Authors:** Ying Yang, Jin Wang, Fei Shi, Aijun Shan, Shihai Xu, Wen Lv

**Affiliations:** 1 Department of Pediatrics, Futian Women and Children Health Institute, Shenzhen 518045, China; 2 Department of Emergency, Shenzhen People’s Hospital (The Second Clinical Medical College, Jinan University, The First Affiliated Hospital, Southern University of Science and Technology), Shenzhen 518020, China

**Keywords:** glioma, BDKRB2, epithelial-to-mesenchymal transition, EMT, prognosis

## Abstract

Bradykinin receptor B2 (BDKRB2) has been reported as an oncogene in several malignancies. In glioma, the role of BDKRB2 remains unknown. This study aimed at investigating its clinical significance and biological function in glioma at the transcriptional level. We selected 301 glioma patients with microarray data from CGGA database and 697 with RNAseq data from TCGA database. Transcriptome and clinical data of 998 samples were analyzed. Statistical analysis and figure generating were performed with R language. BDKRB2 expression showed a positive correlation with the WHO grade of glioma. BDKRB2 was increased in IDH wildtype and mesenchymal subtype of glioma. Gene ontology analysis demonstrated that BDKRB2 was profoundly associated with extracellular matrix organization in glioma. GSEA analysis revealed that BDKRB2 was particularly correlated with epithelial-to-mesenchymal transition (EMT). GSVA analysis showed that BDKRB2 was significantly paralleled with several EMT signaling pathways, including PI3K/AKT, hypoxia, and TGF-β. Moreover, BDKRB2 expression was significantly correlated with key biomarkers of EMT, especially with N-cadherin, snail, slug, vimentin, TWIST1, and TWIST2. Finally, higher BDKRB2 indicated significantly shorter survival for glioma patients. In conclusion, BDKRB2 was associated with more aggressive phenotypes of gliomas. Furthermore, BDKRB2 was involved in the EMT process and could serve as an independent prognosticator in glioma.

## INTRODUCTION

Gliomas are the most common and aggressive primary brain cancers in adult patients [1]. Despite improvements in diagnosis and treatment, the prognosis of glioma remains unfavorable. Especially for glioblastoma (GBM) patients, the most malignant type, the median survival time is only about fifteen months [2, 3]. Epithelial-to-mesenchymal transition (EMT) has been widely reported as a key mechanism in promoting migration, invasion, and tumor progression in glioma [4]. Identification of novel EMT-related markers will facilitate the development of potential molecular targets for glioma patients.

Bradykinin (BK) is a vasoactive peptide produced from kininogen precursor’s cleavage under kallikrein’s action [5]. BK participates in a range of pathophysiological processes, including vasodilation, vascular permeability, pain-sensing, smooth muscle contraction, and inflammation modulation. The BK’s biological function is mediated by the activation of two G protein-coupled receptors: Bradykinin receptor B1 (BDKRB1) and BDKRB2. BDKRB1 is weakly or not expressed under normal physiological conditions, while proinflammatory mediators and oxidative stress can upregulate it, and it usually binds to the active metabolite des-Arg^9^-BK. However, BDKRB2 is constitutively expressed across different tissues, and it has a high affinity with BK [6]. There is a crosstalk between BDKRB1 and BDKRB2. Upregulation of BDKRB1 can be associated with BDKRB2 downregulation [7].

The BK system plays an important role in cancer occurrence and progression [8]. The BK system stimulates cell proliferation, migration, and angiogenesis, contributing to tumor progression [9]. As a vital receptor of bradykinin, BDKRB2 has been widely reported in a range of malignancies, including cervical cancer [10], triple-negative breast cancer [11], hepatocellular carcinoma (HCC) [12, 13], gastric cancer [14–16], colorectal cancer [17], prostate cancer [18], bladder cancer [19], head and neck squamous cell carcinomas [20], and chondrosarcoma [21]. Across different malignancies, via activating BDKRB2, BK promotes tumor progression through various pathways. For example, the BK-BDKRB2 axis can promote angiogenesis by increasing vascular permeability and by upregulating vascular endothelial growth factor (VEGF) in a sarcoma mouse model [22] and a Walker 256 carcinoma cell-bearing rat model [23]. Yu et al. further demonstrated that the BK-BDKRB2 axis activated Akt-mTOR signaling and downstream NF-κB and activator protein 1 (AP-1), which activated VEGF in human prostate cancer cells [24]. In HCC, BK-BDKRB2 promotes the migration and invasion of tumor cells through transient receptor potential cation channel subfamily M member 7 (TRPM7) and matrix metalloproteinase-2 (MMP2) [13]. In melanoma, BK-BDKRB2 upregulates endothelin-1 and subsequently increases the capacity of migration and invasion [25]. In gastric cancer, the BK-BDKRB2 axis promotes cell proliferation, migration, invasion through ERK signaling pathway [14]. Moreover, several studies sought to investigate the correlation between BDKRB2 expression and clinical characterization and have concluded relatively consistent results across different cancers. A higher expression level of BDKRB2 was reported to be correlated with more malignant features and shorter survival [10, 11, 26, 27].

Heretofore, some researchers have investigated the potential biological functions of BDKRB2 in glioma based on *in-vivo* and *in-vitro* experimental studies [7, 28–30]. However, we could not find a systematic report about BDKRB2 expression in pan-glioma from the clinical perspective. In the present study, 998 glioma patients with transcriptome data were enrolled and analyzed, aiming at investigating the clinical significance, characterization of expression profiling, and biological function of BDKRB2 in glioma.

## RESULTS

### BDKRB2 expression was correlated with aggressive phenotypes of glioma

**Figure 1:**
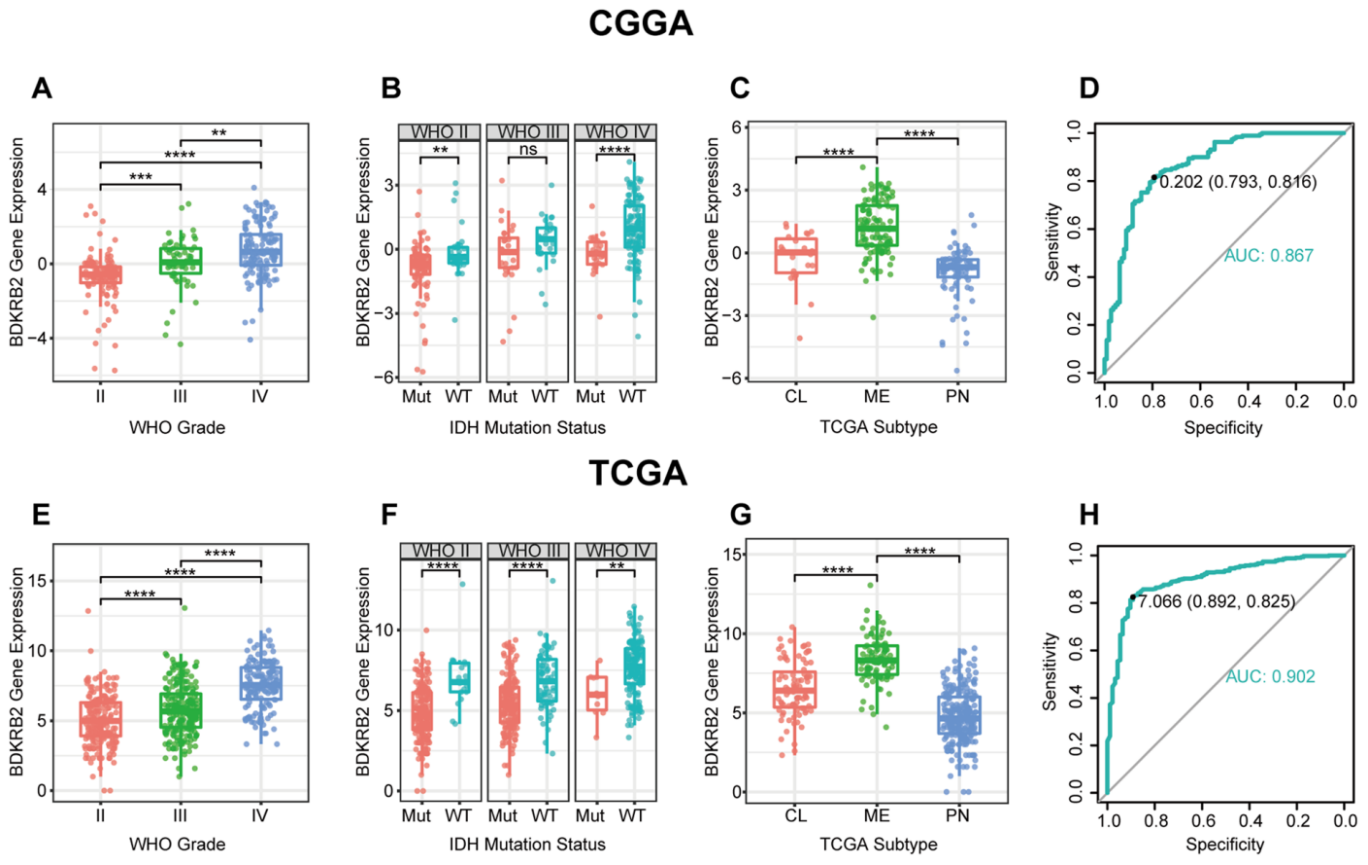
BDKRB2 expression in CGGA and TCGA dataset according to WHO grade (**A**, **E**), IDH mutation status (**B**, **F**), TCGA molecular subtype (**C**, **G**) and ROC curves (**D**, **H**) for distinguishing mesenchymal subtype. * indicates p value < 0.05, **indicates p value < 0.01, *** indicates p value < 0.001, **** indicates p value < 0.0001.

BDKRB2 expression levels were compared across different WHO grades. The results of both CGGA and TCGA cohorts consistently showed a significant positive correlation between BDKRB2 expression and WHO grade (Figure 1A, 1E). In addition, when patients were subclassified with respect to IDH mutation status, IDH wildtype was found to be more associated with an increased pattern of BDKRB2 expression in both datasets, except for the subgroup of WHO grade II in CGGA dataset, which also exhibited an apparent trend (Figure 1B, 1F). These results suggested that higher BDKRB2 was paralleled with higher malignancy in glioma. Moreover, the correlation between BDKRB2 and TCGA molecular subtype was further examined. As shown in Figure 1C, 1G, BDKRB2 expression in mesenchymal subtype were significantly upregulated than that in other subtypes, suggesting that BDKRB2 expression could contribute as a specific marker for the mesenchymal subtype. ROC curves were subsequently performed to evaluate the performance of BDKRB2 in distinguishing mesenchymal subtype. Areas under curves (AUC) were 86.7% in CGGA and 90.2% in TCGA, respectively (Figure 1D, 1H).

Many other genes have been identified as the mesenchymal subtype’s biomarkers, such as PD1 [31], PD-L1 [32], TIM3 [33], CD163 [34], PSAP [35], and UPP1 [36]. These biomarkers were concurrently enrolled in ROC analysis to assess the sensitivity and specificity of BDKRB2. As shown in Supplementary Figure 1, BDKRB2 was comparable to other molecules reported previously and seemed superior to most of them in both CGGA and TCGA.

### BDKRB2-related biological process

**Figure 2:**
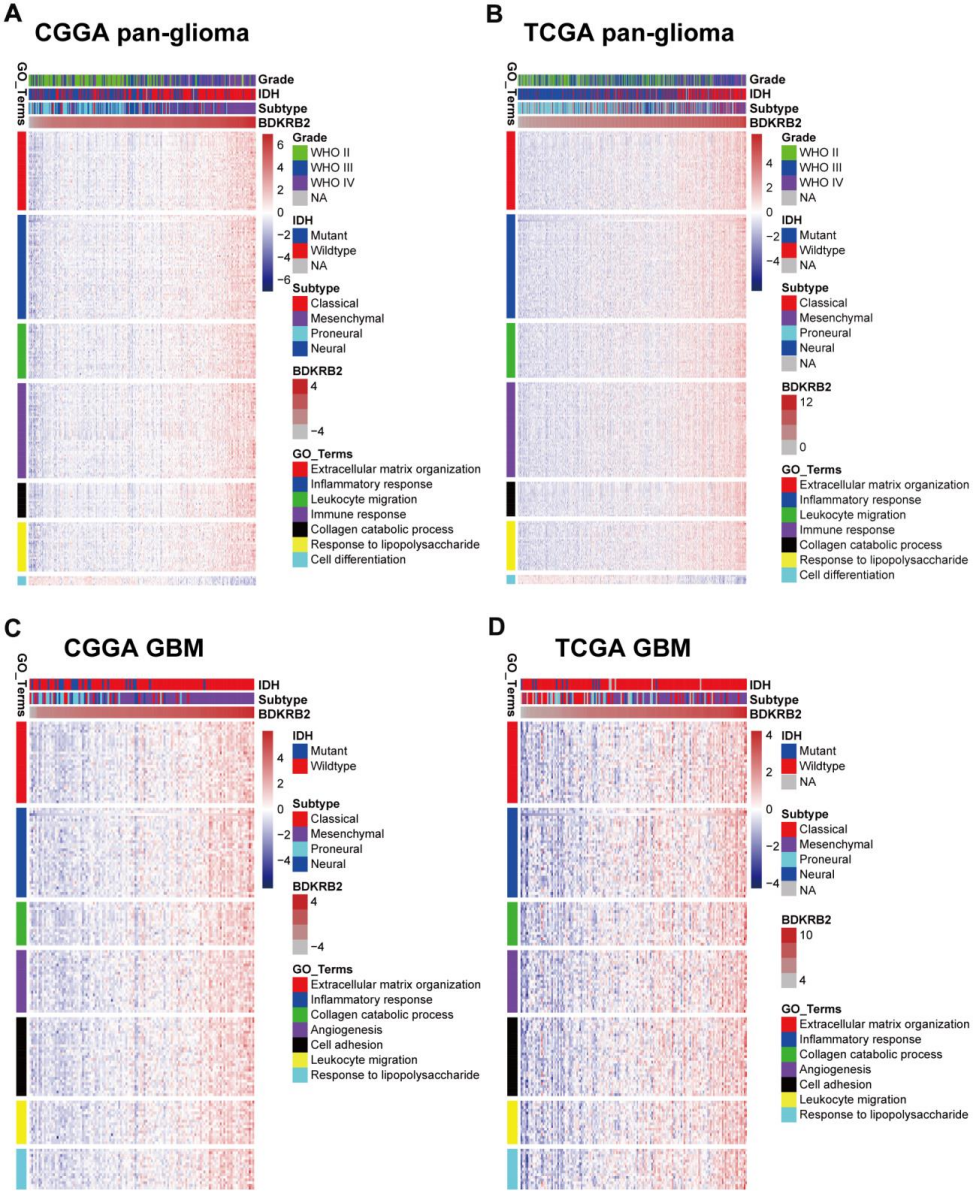
Gene Ontology analysis for BDKRB2 in pan-glioma (**A**, **B**) and glioblastoma (**C**, **D**).

To explore the biological process of BDKRB2 in glioma, Pearson correlation test was performed between BDKRB2 and other genes. With the criteria of Pearson coefficient |r| > 0.5, we identified 1011 BDKRB2-positively-correlated genes and 338 BDKRB2-negatively-correlated genes in CGGA, and 911 BDKRB2-positively-correlated genes and 69 BDKRB2-negatively-correlated genes in TCGA. To ensure accuracy, BDKRB2-significantly-correlated genes that were overlapped between both datasets were selected for GO analysis. Venn diagrams (Supplementary Figure 2) were constructed, illustrating overlap of 555 BDKRB2-positively-correlated genes and 20 BKDRB2-negatively-correlated genes (Supplementary Table 1), which were subsequently annotated in GO analysis. We found that BDKRB2-positively-correlated genes were mainly involved in extracellular matrix organization (*p* = 1.53E-23), inflammatory response (*p* = 1.29E-22), leukocyte migration (*p* = 6.21E-18), immune response (*p* = 6.30E-17), collagen catabolic process (*p* = 5.80E-13) and response to lipopolysaccharide (*p* = 8.58E-12), suggesting that BDKRB2 was tightly associated with EMT process and inflammatory response in glioma. As expected, BDKRB2-negatively-correlated genes were largely associated with normal biological processes, such as cell differentiation (*p* = 0.001) (Figure 2A, 2B and Supplementary Table 3).

Given GBM as a distinct subgroup of glioma, we then conducted an independent GO enrichment analysis in this group. In GBM of both datasets, the Venn diagram (Supplementary Figure 3) exhibited an overlap of 257 BDKRB2-positively-correlated genes (Supplementary Table 2). They were found to reveal a similar biological process pattern as those in pan-glioma (Figures 2C, 2D and Supplementary Table 3). Besides, it should be noted that BDKRB2 showed a positive correlation with angiogenesis (*p* = 2.12E-12), which was paralleled with the characterization of vascularization in GBM.

### BDKRB2 was associated with EMT

**Figure 3:**
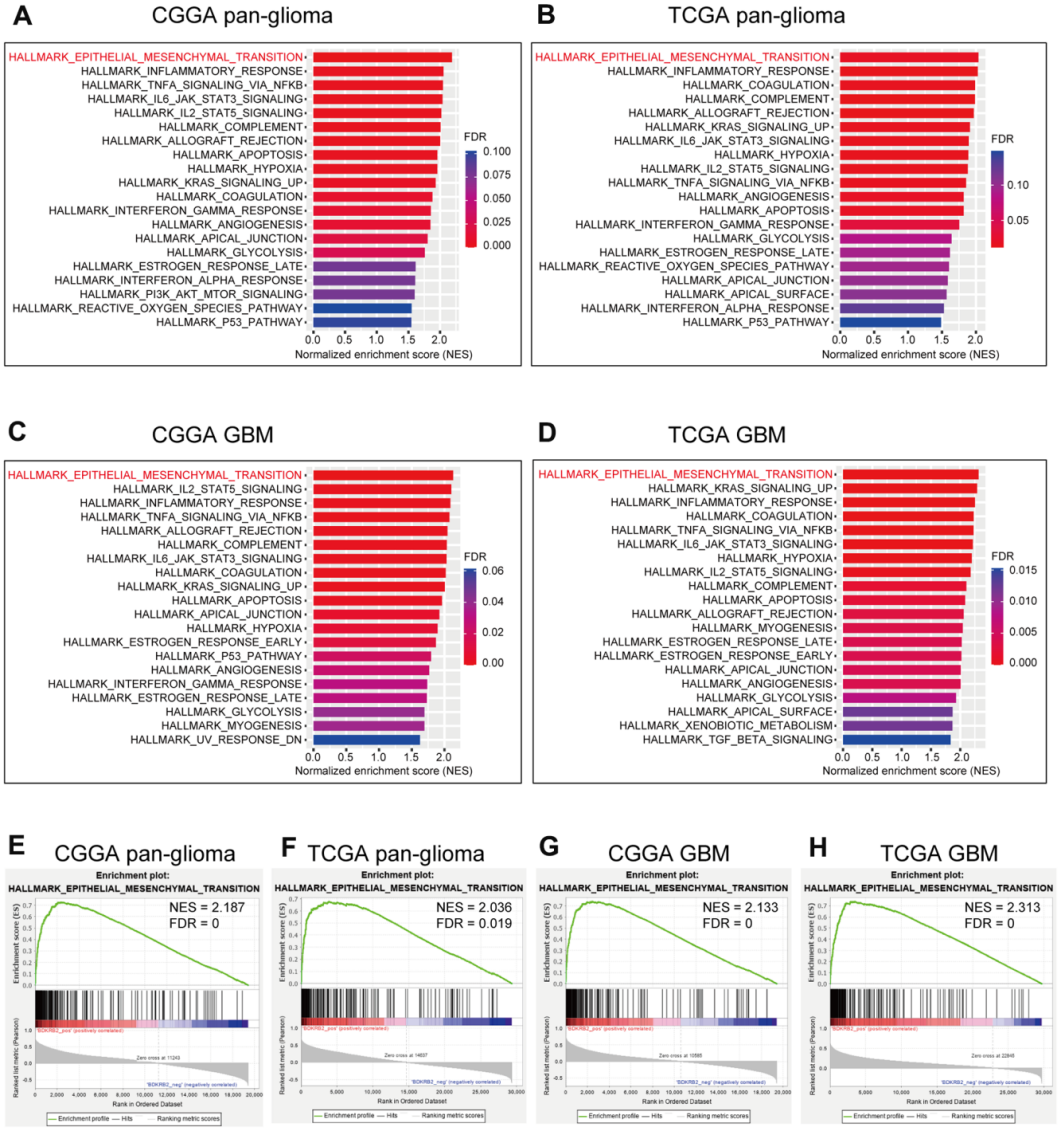
GSEA of BDKRB2 in pan-glioma (**A**, **B**) and glioblastoma (**C**, **D**), and GSEA plots for EMT enrichment according to BDKRB2 expression in pan-glioma (**E**, **F**) and glioblastoma (**G**, **H**).

To further illustrate the biological process of BDKRB2 in glioma, GSEA analyses were performed in both CGGA and TCGA datasets. We found that BDKRB2 was most correlated with EMT in CGGA (NES = 2.187, FDR = 0) (Figure 3A, 3E), which was further validated in TCGA (NES = 2.036, FDR = 0.019) (Figure 3B, 3F). Furthermore, GSEA analysis showed a similar pattern of functional enrichment in GBM of both datasets (Figure 3C, 3D, 3G, 3H). These results indicated that BDKRB2 was profoundly associated with EMT phenotype in glioma.

### BDKRB2-related EMT signaling pathways

**Figure 4:**
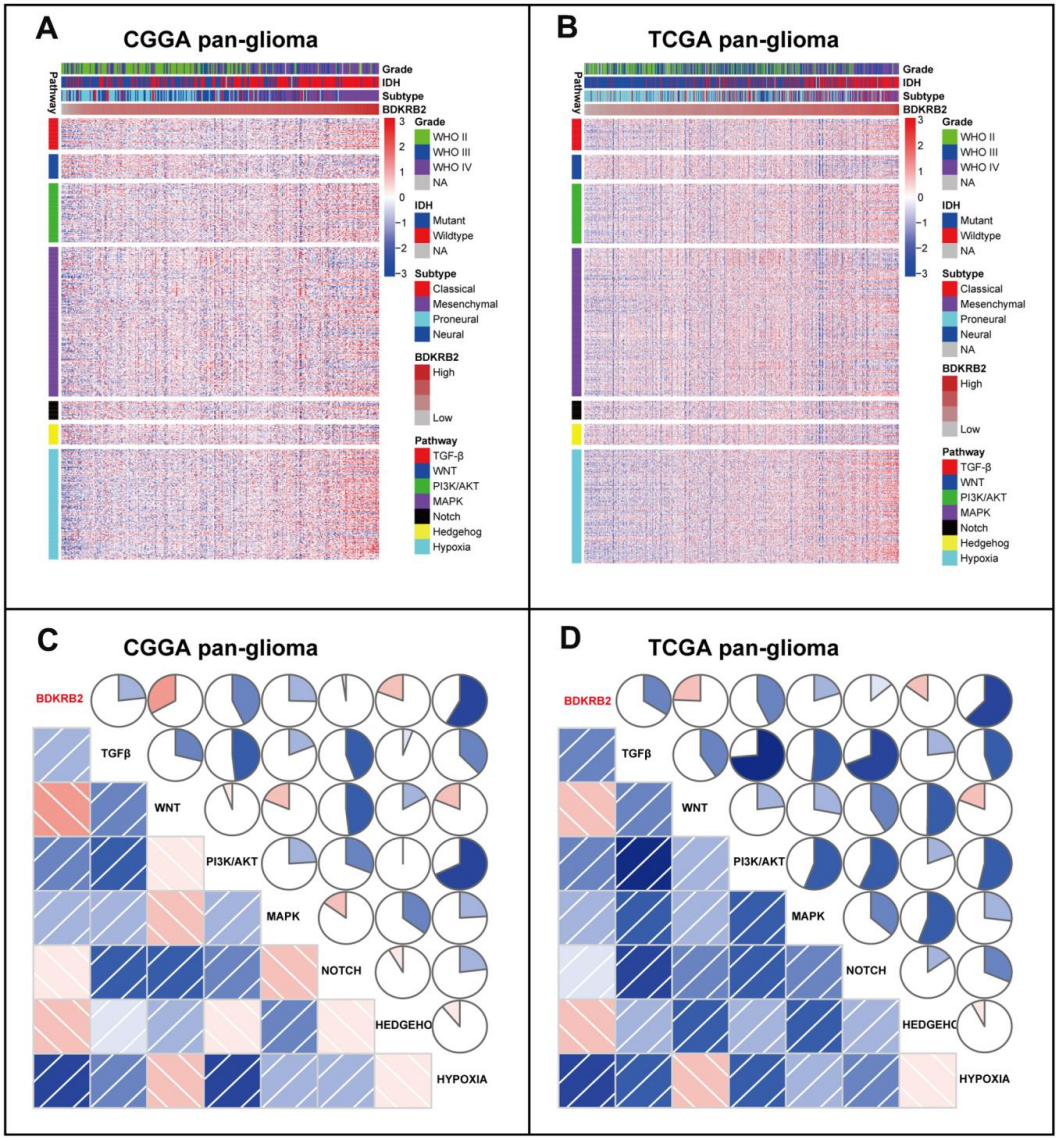
Cluster (**A**, **B**) and GSVA (**C**, **D**) of BDKRB2-related EMT signaling pathways in pan-glioma. In **C** and **D**, the blue and red colors represent positive and negative correlation, respectively. A darker color and a bigger sectorial area represent a higher correlation coefficient.

To further investigate the relationship between BDKRB2 and EMT, we downloaded seven gene sets from the GSEA website (Supplementary Table 4), which were subsequently transformed into metagenes, representing different EMT-related signaling pathways, summarized by Gonzalez et al. [37]. As shown in Figures 4A, 4B, three clusters, including TGF-β, PI3K/AKT, and hypoxia signaling pathway, were significantly associated with BDKRB2 expression. To quantify what we observed in clusters, Gene Set Variation Analysis (GSVA) was performed to generate seven metagenes based on corresponding genes of seven EMT-related signaling pathways. According to Pearson r-value between BDKRB2 and seven metagenes, Corrgrams were generated to evaluate their intercorrelations (Figure 4C, 4D). BDKRB2 showed a robust correlation with TGF-β, PI3K/AKT, and hypoxia signaling pathway, while only showed a weak correlation with WNT, MAPK, NOTCH, and HEDGEHOG pathway, consistent with what we observed in Figure 4A, 4B. Moreover, taking GBM as a distinct group, we investigated BDKRB2-related EMT signaling pathways in the GBM subgroup. It turned out that, other than TGF-β, PI3K/AKT, and hypoxia, BDKRB2 also showed a robust correlation with MAPK signaling pathway (Supplementary Figure 4).

### BDKRB2 was associated with EMT biomarkers

**Figure 5:**
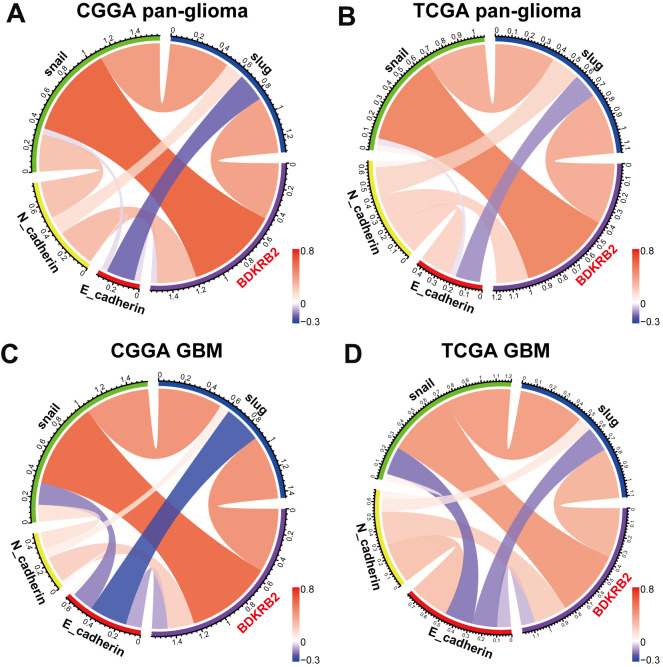
Correlation of BDKRB2 and key EMT biomarkers in pan-glioma (**A**, **B**) and glioblastoma (**C**, **D**).

To further validate the role of BDKRB2 in the EMT signaling pathway, we examined the correlation between BDKRB2 and EMT biomarkers, including E-cadherin, N-cadherin, snail, and slug. Circos plots revealed that BDKRB2 expression was significantly associated with N-cadherin, snail, and slug (Figure 5A, 5B). Pearson correlation tests were additionally performed in GBM. As shown in Figure 5C, 5D, the correlation between BDKRB2 and these markers in GBM was also very robust in both datasets. While the correlation between BDKRB2 and E-cadherin was very weak, which might be deemed as a noise.

Many other biomarkers have been identified as EMT-related targets in EMT [38]. We additionally enrolled EMT-related markers, including β-catenin, vimentin, TWIST1, and TWIST2, and put them into analysis together with BDKRB2. Subsequent Circos plots revealed that BDKRB2 expression was especially correlated with vimentin, TWIST1, and TWIST2 (Supplementary Figure 5).

### Higher BDKRB2 predicted shorter survival for glioma

**Figure 6:**
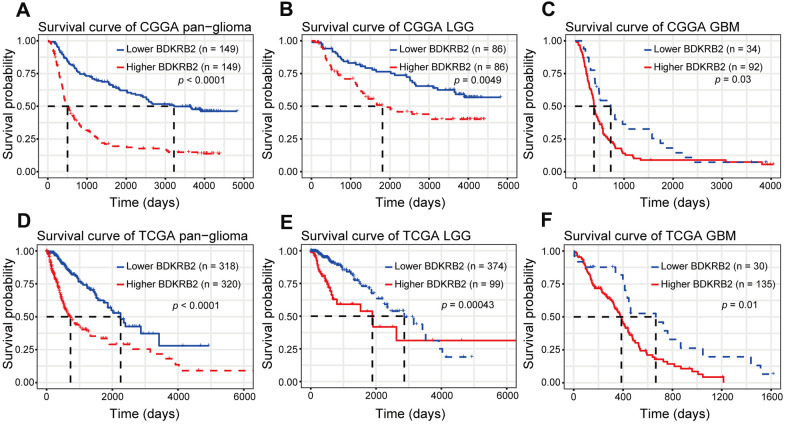
Survival analysis for BDKRB2 in pan-glioma (**A**, **D**), LGG (**B**, **E**) and GBM (**C**, **F**).

Kaplan-Meier (KM) survival analyses were performed to examine the prognostic role of BDKRB2 in glioma. According to BDKRB2 expression, pan-glioma samples were divided into two groups in each dataset. As shown in Figure 6A, 6D, a higher level of BDKRB2 expression predicted a significantly shorter survival. Moreover, a similar KM survival curve pattern was observed among patients with LGG (Figures 6B, 6E) and GBM (Figure 6C, 6F). To identify the independent effect of BDKRB2 on glioma prognosis, Cox regression analyses were performed with covariates, including BDKRB2 expression, age, and WHO grade. Multivariate analyses revealed that BDKRB2 expression was a significant prognosticator independent of age and WHO grade in both CGGA and TCGA (Table 1).

**Table 1:** Cox regression analysis of overall survival in glioma.

**Covariates**	**CGGA_301**						**TCGA**				
	**Univariate**			**Multivariate**			**Univariate**			**Multivariate**	
	**HR(95% CI)**	***P***		**HR(95% CI)**	***P***		**HR(95% CI)**	***P***		**HR(95% CI)**	***P***
Age	1.041 (1.027-1.055)	0.000		1.018 (1.005-1.1.031)	0.008		1.075 (1.062-1.087)	0.000		1.048 (1.034-1.062)	0.000
Grade	2.670 (2.221-3.210)	0.000		2.329 (1.905-2.847)	0.000		5.057 (3.915-6.532)	0.000		3.011 (2.255-4.019)	0.000
BDKRB2	1.420 (1.282-1.573)	0.000		1.126 (1.012-1.253)	0.030		1.443 (1.335-1.559)	0.000		1.155 (1.055-1.265)	0.002

## DISCUSSION

Emerging evidence indicates BDKRB2 as a pivotal target in tumorigenesis. BDKRB2 as a frequently amplified molecule has been observed in a range of cancers, including cervical cancer [10], triple-negative breast cancer [11], hepatocellular carcinoma (HCC) [12, 13], gastric cancer [14–16], colorectal cancer [17], prostate cancer [18], bladder cancer [19], head and neck squamous cell carcinomas [20], and chondrosarcoma [21]. In glioma, only a few studies have reported that BDKRB2 was dysregulated in GBM cell lines [7, 28–30]. However, the expression profile and prognostic value of BDKRB2 in glioma are still largely unknown.

In the present study, we investigated the transcriptional expression profiles of BDKRB2 in 998 glioma patients and revealed that BDKRB2 expression showed a significantly positive correlation with the WHO grade of glioma. Furthermore, higher BDKRB2 expression was usually accompanied by a more aggressive and malignant phenotype in glioma, including GBM, IDH wildtype, and mesenchymal subtype. Moreover, higher BDKRB2 expression indicated a significantly shorter survival for patients with glioma across different WHO grades. These findings suggested that BDKRB2 played a vital role in the malignant progression of glioma, in line with other malignancies reported previously. Understanding the molecular mechanism of BDKRB2 in glioma may provide a novel therapeutic target to overcome this fatal disease.

To elucidate the biological function of BDKRB2 in glioma, we further performed GO and GSEA analysis. GO analysis revealed that BDKRB2 was highly associated with extracellular matrix organization and collagen catabolic process in both pan-glioma and GBM, suggesting that glioma cells through their interactions with BDKRB2 might acquire functions that enhance matrix remodeling, cell migration, invasion, and tumor progression, consistent with the results presented by Montana et al. [28]. They concluded that the activation of BDKRB2 in glioma cells caused intracellular Ca^2+^ oscillations and subsequently enhanced glioma cell migration/invasion. In other types of malignancies, BDKRB2 also exhibited the biological function of promoting cell migration, invasion, and metastasis in hepatocellular carcinoma [12, 13], gastric cancer [14, 16], colorectal cancer [17], prostate cancer [18], head and neck squamous cell carcinoma [20] and chondrosarcoma [21]. In addition, GSEA analyses revealed remarkable evidence that BDKRB2 expression was particularly correlated with EMT, which had been extensively confirmed to play a key role not only in glioma migration/invasion but also in glioma recurrence and therapeutic resistance [39–41]. These results enlightened us that BDKRB2 might promote tumorigenesis and glioma progression mainly through modulating the EMT signaling pathway. Besides, GO and GSEA also revealed that BDKRB2 played a vital role in the tumor-induced inflammatory response in both pan-glioma and GBM subgroup, which might be another mechanism for the oncogenic role of BDKRB2 in glioma.

To further validate the role of BDKRB2 in the glioma EMT process, we selected a series of EMT-related signaling pathways and biomarker, which were then analyzed to determine their interaction with BDKRB2. We found that BDKRB2 expression showed a robust correlation with PI3K/AKT, hypoxia, and TGF-β signaling pathway, suggesting that BDKRB2 might promote the EMT process through these pathways. Moreover, most EMT biomarkers, including N-cadherin, snail, slug, vimentin, TWIST1, and TWIST2, were significantly correlated with BDKRB2, which suggested that BDKRB2 might profoundly interact with these key molecules of EMT, further confirming the involvement of BDKRB2 in glioma EMT. Thus, our findings might bring a novel EMT target for potential glioma treatment.

In conclusion, the present study demonstrated that BDKRB2 expression was associated with more malignant glioma phenotypes and predicted much worse survival for patients. Moreover, BDKRB2 was significantly associated with the EMT process. However, a limitation of the current study was that no experimental validation was performed. Further *in-vitro* and *in-vivo* studies are needed to validate its role in glioma.

## MATERIALS AND METHODS

### Sample and data collection

Transcriptome and clinical data of glioma patients were available on Chinese Glioma Genome Atlas (CGGA) website (http://www.cgga.org.cn/) [42, 43] and TCGA website (http://cancergenome.nih.gov/) [44]. A total of 998 glioma patients, including 301 CGGA microarray data (GeneSpring GX 11.0 normalization) and 697 TCGA RNAseq data (RSEM normalization, level 3), were enrolled. The baseline characteristics of patients in both cohorts were described in Supplementary Table 5. This study was based on two large public databases, with no use of personally identifiable information. The ethics approval was waived by the Ethics Committee of Shenzhen People’s Hospital.

### Statistical analysis

For TCGA cohort, RSEM RNAseq data were log2 transformed. For CGGA cohort, microarray data (already normalized and centered by data provider) were directly analyzed. Statistical analysis was performed with R language. Multiple R packages, including ggplot2, pROC, pheatmap, corrgram, circlize, and survival, were used to generate figures. Cox proportional hazard regression analyses were performed with coxph function of survival package. The biological processes of BDKRB2-related genes were annotated using Gene Ontology (GO) (DAVID, https://david.ncifcrf.gov/) enrichment analysis. The hallmarks.all.v7.1.symbols.gmt gene set was selected for Gene Set Enrichment Analysis (GSEA, http://software.broadinstitute.org/). All 301 samples in CGGA and 697 in TCGA were included in pan-glioma GSEA analysis, and 128 GBM samples in CGGA and 167 in TCGA were included in GBM GSEA analysis. The number of permutations was 1000. The enrichment statistic was set as weighted, and the metric for ranking genes was set as Pearson. All statistical tests were two-sided, and a *p*-value of < 0.05 indicated a statistical significance.

## Supplementary Material

Supplementary Figures

Supplementary Table 1

Supplementary Table 2

Supplementary Table 3

Supplementary Table 4

Supplementary Table 5
