# Supplementary material for: BDKRB2 is a novel EMT-related biomarker and predicts poor survival in glioma
Source: Aging (Albany NY). 2021 Mar 3;13(5):7499–516. doi: 10.18632/aging.202614 (PMC7993731; doi:10.18632/aging.202614)
Supplement: Supplementary Figures [file aging-13-202614-s001.pdf]

SUPPLEMENTARY FIGURES

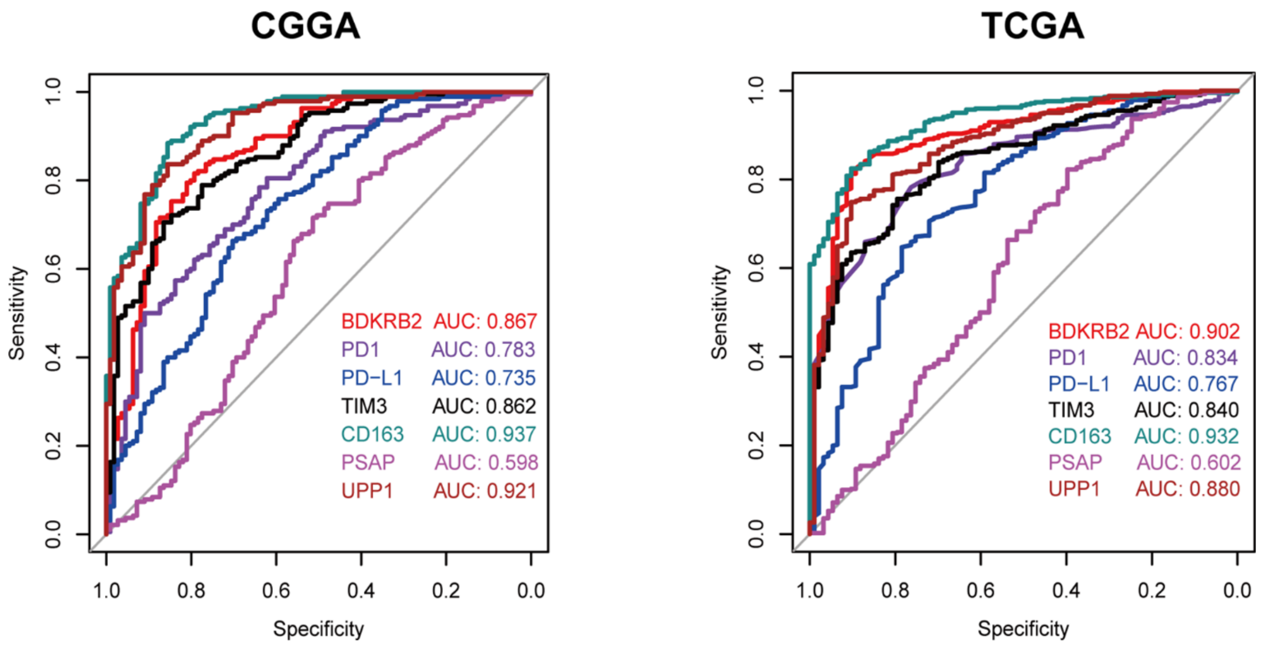

Supplementary Figure 1. Comparison of the diagnostic performance for mesenchymal subtype between BDKRB2 and other biomarkers.

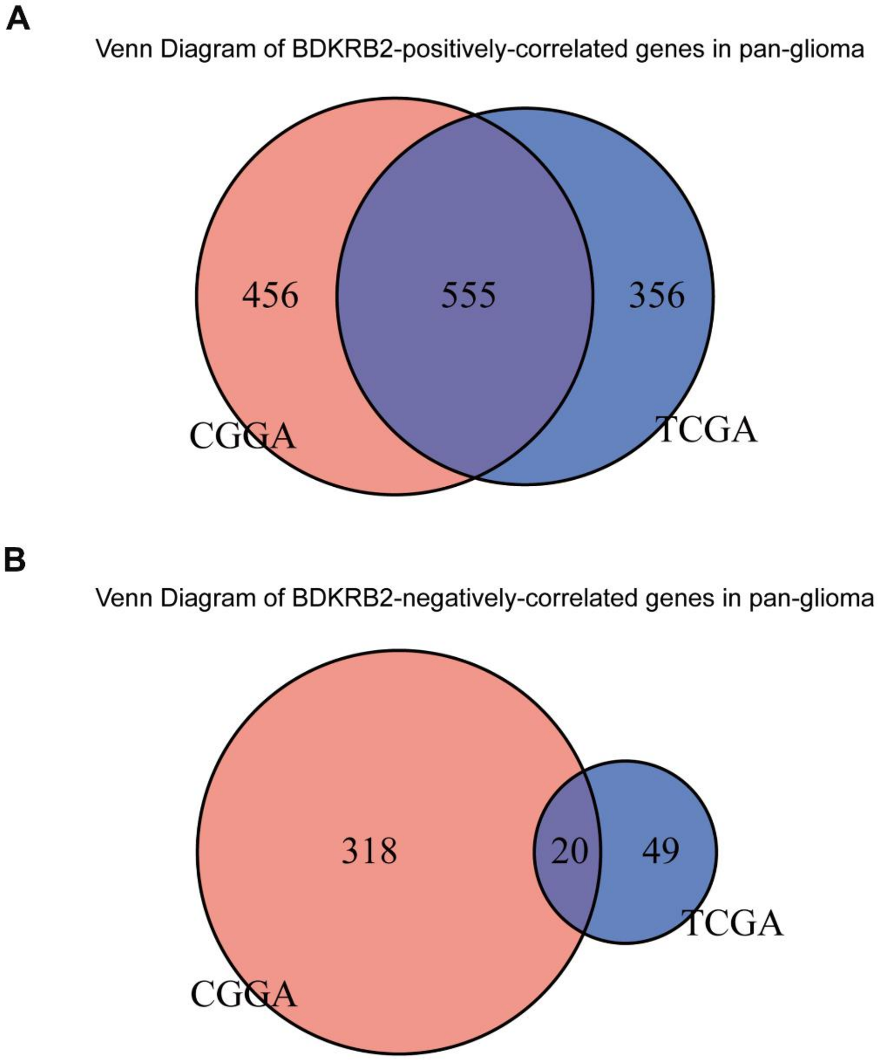

Supplementary Figure 2. Venn Diagram of BDKRB2-significantly (**A** positively; **B** negatively)-correlated genes in pan-glioma.

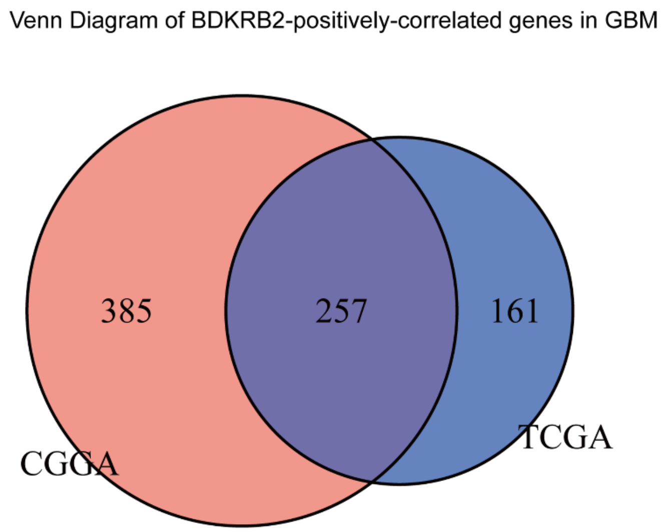

Supplementary Figure 3. Venn Diagram of BDKRB2-positively-correlated genes in glioblastoma.

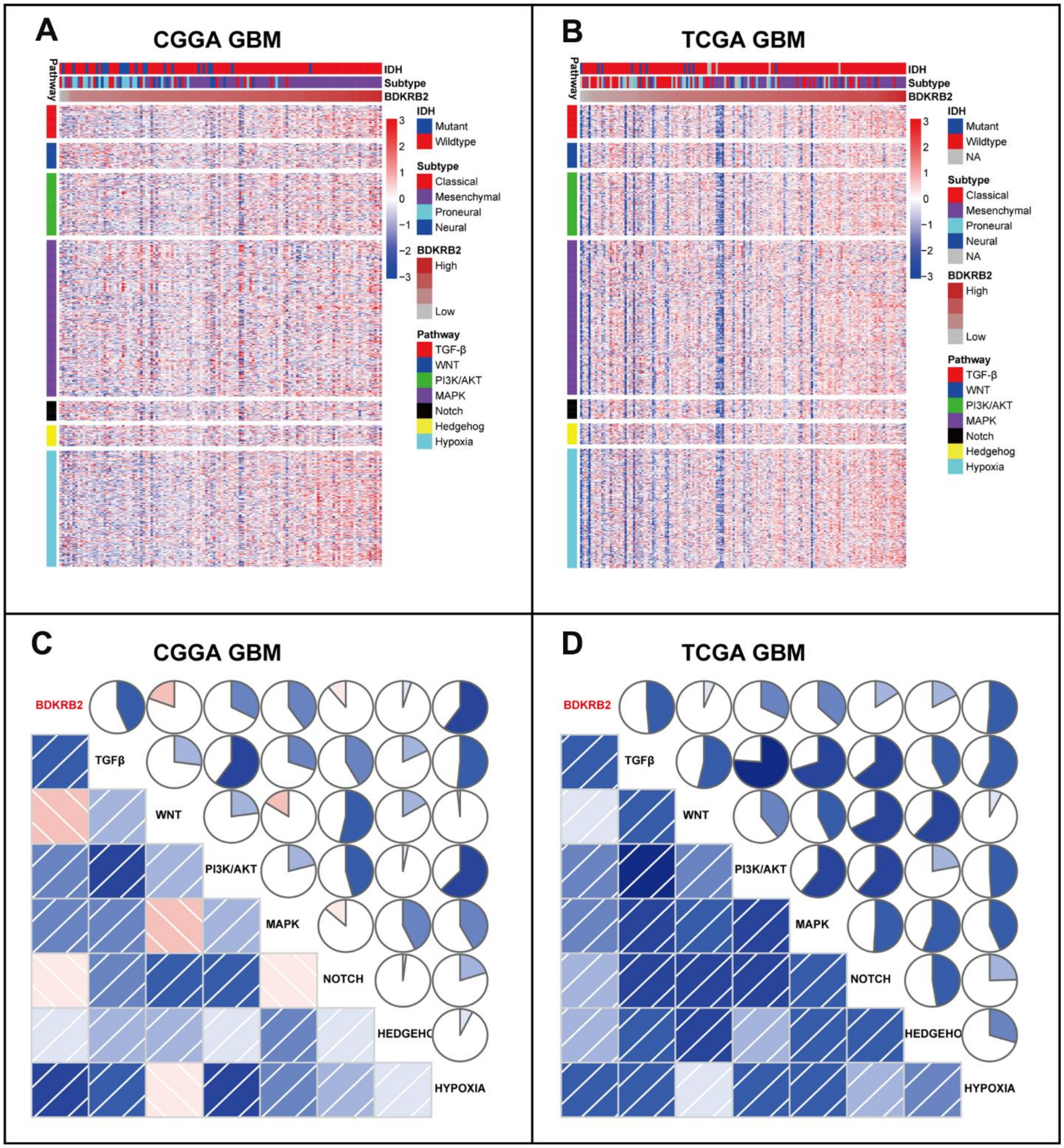

Supplementary Figure 4. Cluster (**A**, **B**) and GSVA (**C**, **D**) of BDKRB2-related EMT signaling pathways in glioblastoma. In **C**, **D**, the blue and red colors represent positive and negative correlation, respectively. A darker color and a bigger sectorial area represent a higher correlation coefficient.

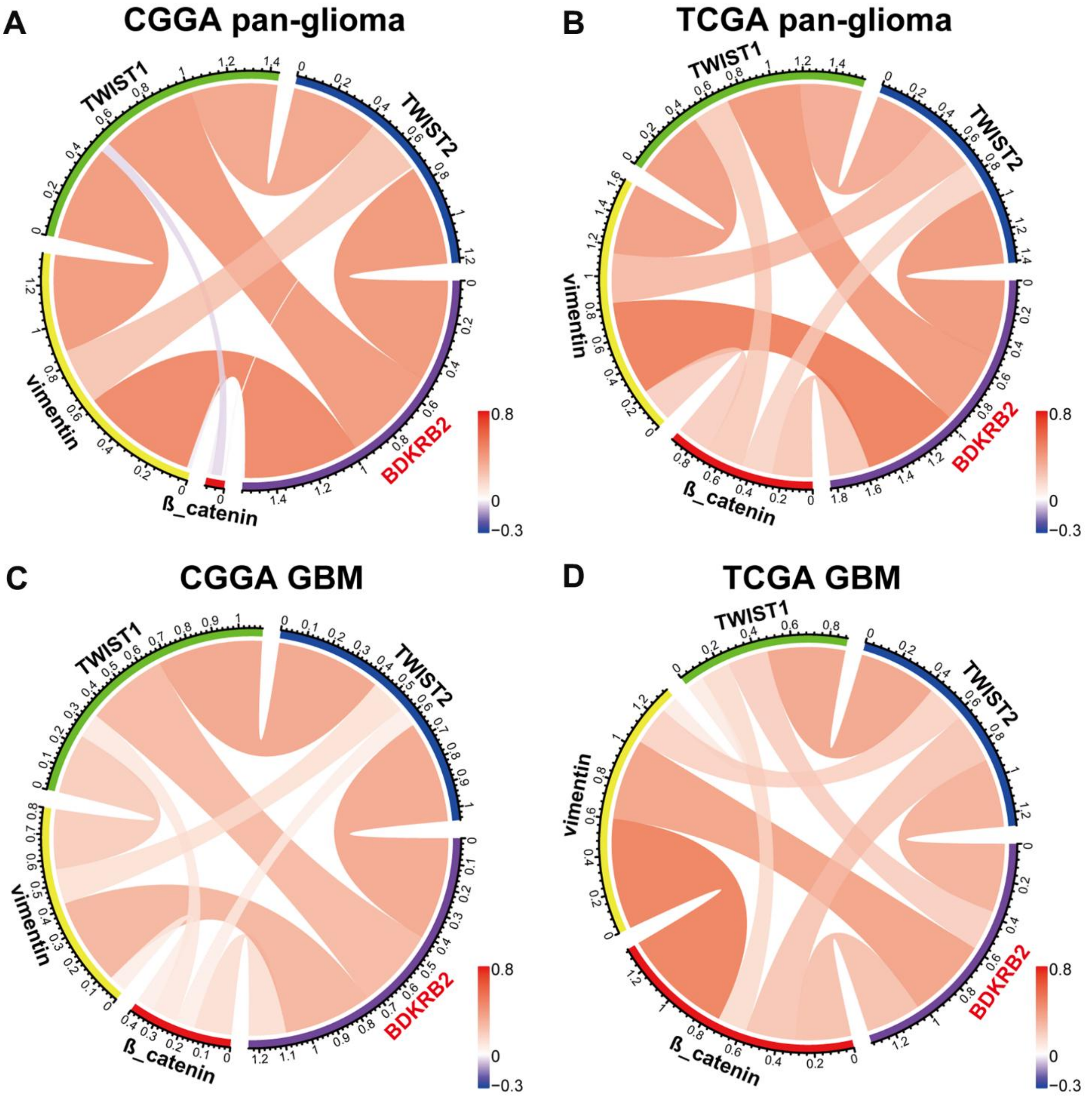

Supplementary Figure 5. Correlation of BDKRB2 and other EMT biomarkers in pan-glioma (**A**, **B**) and glioblastoma (**C**, **D**).
