# Supplementary material for: BDKRB2 is a novel EMT-related biomarker and predicts poor survival in glioma
Source: Aging (Albany NY). 2021 Mar 3;13(5):7499–516. doi: 10.18632/aging.202614 (PMC7993731; doi:10.18632/aging.202614)
Supplement: Supplementary Table 1 [file aging-13-202614-s002.docx]

**Supplementary Table 1. Gene list for BDKRB2-significantly-correlated genes that overlap between CGGA and TCGA pan-glioma.**

***BDKRB2-positively-correlated genes***

| 1 | ABCC3 |
| --- | --- |
| 2 | ACPP |
| 3 | ACTA2 |
| 4 | ACTG2 |
| 5 | ACTN1 |
| 6 | ADAM12 |
| 7 | ADAMTS1 |
| 8 | ADM |
| 9 | AHNAK2 |
| 10 | AHR |
| 11 | AKAP12 |
| 12 | ALOX5 |
| 13 | ALOX5AP |
| 14 | AMIGO2 |
| 15 | ANG |
| 16 | ANXA1 |
| 17 | ANXA2 |
| 18 | AOX1 |
| 19 | AP1S3 |
| 20 | AP2S1 |
| 21 | APCDD1L |
| 22 | APOBEC3C |
| 23 | APOBEC3F |
| 24 | APOBEC3G |
| 25 | APOL1 |
| 26 | AQP9 |
| 27 | ARHGAP18 |
| 28 | ARHGAP9 |
| 29 | ARHGDIB |
| 30 | ARID5A |
| 31 | ARL4C |
| 32 | ARNTL2 |
| 33 | ARPC1B |
| 34 | ARPC2 |
| 35 | ARPC5 |
| 36 | ASPN |
| 37 | ASS1 |
| 38 | ATF3 |
| 39 | ATF5 |
| 40 | ATP2A3 |
| 41 | B3GNT5 |
| 42 | BACE2 |
| 43 | BATF |
| 44 | BATF3 |
| 45 | BCAT1 |
| 46 | BCL2A1 |
| 47 | BCL3 |
| 48 | BDKRB1 |
| 49 | BDKRB2 |
| 50 | BGN |
| 51 | BHLHE40 |
| 52 | BIRC3 |
| 53 | BST1 |
| 54 | C15orf48 |
| 55 | C1orf54 |
| 56 | C1R |
| 57 | C1RL |
| 58 | C1S |
| 59 | C2 |
| 60 | C5AR1 |
| 61 | C6orf141 |
| 62 | CA13 |
| 63 | CA9 |
| 64 | CALHM2 |
| 65 | CALU |
| 66 | CAPG |
| 67 | CAPZA1 |
| 68 | CARD16 |
| 69 | CASP1 |
| 70 | CASP4 |
| 71 | CASP5 |
| 72 | CASP7 |
| 73 | CASP8 |
| 74 | CAST |
| 75 | CATSPER1 |
| 76 | CAV1 |
| 77 | CAV2 |
| 78 | CCL2 |
| 79 | CCL20 |
| 80 | CCL5 |
| 81 | CCL7 |
| 82 | CCR2 |
| 83 | CCR5 |
| 84 | CD14 |
| 85 | CD163 |
| 86 | CD2 |
| 87 | CD247 |
| 88 | CD248 |
| 89 | CD274 |
| 90 | CD276 |
| 91 | CD3D |
| 92 | CD40 |
| 93 | CD44 |
| 94 | CD52 |
| 95 | CD58 |
| 96 | CD6 |
| 97 | CD63 |
| 98 | CD70 |
| 99 | CD80 |
| 100 | CD8A |
| 101 | CD93 |
| 102 | CD96 |
| 103 | CDCP1 |
| 104 | CDR2 |
| 105 | CELSR1 |
| 106 | CFB |
| 107 | CFH |
| 108 | CFI |
| 109 | CHI3L1 |
| 110 | CHI3L2 |
| 111 | CHPF2 |
| 112 | CHRNA9 |
| 113 | CIITA |
| 114 | CLCF1 |
| 115 | CLDN23 |
| 116 | CLEC2B |
| 117 | CLEC5A |
| 118 | CLIC1 |
| 119 | CNN1 |
| 120 | COL15A1 |
| 121 | COL1A1 |
| 122 | COL1A2 |
| 123 | COL3A1 |
| 124 | COL4A1 |
| 125 | COL4A2 |
| 126 | COL5A1 |
| 127 | COL5A2 |
| 128 | COL6A2 |
| 129 | COL6A3 |
| 130 | COL8A1 |
| 131 | CP |
| 132 | CST6 |
| 133 | CST7 |
| 134 | CSTA |
| 135 | CTBS |
| 136 | CTHRC1 |
| 137 | CTSB |
| 138 | CTSC |
| 139 | CTSZ |
| 140 | CXCL10 |
| 141 | CXCL6 |
| 142 | CYB561 |
| 143 | CYTIP |
| 144 | DEDD2 |
| 145 | DENND2D |
| 146 | DES |
| 147 | DIRAS3 |
| 148 | DOK2 |
| 149 | DOK3 |
| 150 | DPYD |
| 151 | DUSP23 |
| 152 | DUSP5 |
| 153 | DYNLT3 |
| 154 | DYRK3 |
| 155 | EFEMP2 |
| 156 | EFNB2 |
| 157 | EGFL6 |
| 158 | EHD2 |
| 159 | EHD4 |
| 160 | ELF4 |
| 161 | ELK3 |
| 162 | EMB |
| 163 | EMILIN1 |
| 164 | EMILIN2 |
| 165 | EMP1 |
| 166 | EMP3 |
| 167 | ENG |
| 168 | ESM1 |
| 169 | EVC2 |
| 170 | F11R |
| 171 | F13A1 |
| 172 | F3 |
| 173 | FABP5 |
| 174 | FAM114A1 |
| 175 | FAM20A |
| 176 | FAM20C |
| 177 | FAS |
| 178 | FBLIM1 |
| 179 | FBLN5 |
| 180 | FBLN7 |
| 181 | FBP1 |
| 182 | FCER1G |
| 183 | FCGR2A |
| 184 | FCGR2B |
| 185 | FCGR3A |
| 186 | FES |
| 187 | FHL2 |
| 188 | FHL3 |
| 189 | FHOD1 |
| 190 | FILIP1L |
| 191 | FKBP9 |
| 192 | FLNA |
| 193 | FLNC |
| 194 | FMOD |
| 195 | FN1 |
| 196 | FNDC3B |
| 197 | FOSL1 |
| 198 | FOSL2 |
| 199 | FPR1 |
| 200 | FPR2 |
| 201 | FSTL1 |
| 202 | FTL |
| 203 | FUCA1 |
| 204 | FUCA2 |
| 205 | G0S2 |
| 206 | GADD45A |
| 207 | GAL |
| 208 | GALNT5 |
| 209 | GAPT |
| 210 | GBP1 |
| 211 | GBP2 |
| 212 | GBP5 |
| 213 | GCLM |
| 214 | GCNT1 |
| 215 | GDF15 |
| 216 | GLIPR1 |
| 217 | GLRX |
| 218 | GMFG |
| 219 | GNA15 |
| 220 | GNG12 |
| 221 | GPR157 |
| 222 | GPR65 |
| 223 | GPR84 |
| 224 | GPRC5A |
| 225 | GPX8 |
| 226 | GZMA |
| 227 | HAMP |
| 228 | HEXB |
| 229 | HFE |
| 230 | HGF |
| 231 | HK3 |
| 232 | HLA-DMA |
| 233 | HLA-DOA |
| 234 | HLA-DRB1 |
| 235 | HMOX1 |
| 236 | HRH1 |
| 237 | HS3ST3B1 |
| 238 | HSPA6 |
| 239 | HSPG2 |
| 240 | HTRA3 |
| 241 | IBSP |
| 242 | ICAM1 |
| 243 | IER5L |
| 244 | IFI30 |
| 245 | IFITM2 |
| 246 | IFITM3 |
| 247 | IFNGR2 |
| 248 | IGFBP2 |
| 249 | IGFBP6 |
| 250 | IKBIP |
| 251 | IL10 |
| 252 | IL15 |
| 253 | IL1R1 |
| 254 | IL1RN |
| 255 | IL2RA |
| 256 | IL2RB |
| 257 | IL4I1 |
| 258 | IL4R |
| 259 | IL6 |
| 260 | IL7R |
| 261 | IMPA2 |
| 262 | IQGAP1 |
| 263 | IRF1 |
| 264 | ISG20 |
| 265 | ISLR |
| 266 | ITGA3 |
| 267 | ITGA4 |
| 268 | ITGA5 |
| 269 | ITGB1 |
| 270 | ITGB3 |
| 271 | ITK |
| 272 | ITPKC |
| 273 | JAK3 |
| 274 | KCNE3 |
| 275 | KCNJ15 |
| 276 | KCNN4 |
| 277 | KDELR1 |
| 278 | KDELR3 |
| 279 | KIAA0040 |
| 280 | KLHDC7B |
| 281 | KMO |
| 282 | KYNU |
| 283 | LAMB1 |
| 284 | LAMC1 |
| 285 | LATS2 |
| 286 | LCP2 |
| 287 | LDHA |
| 288 | LGALS1 |
| 289 | LGALS3 |
| 290 | LGALS8 |
| 291 | LIF |
| 292 | LILRB3 |
| 293 | LOX |
| 294 | LOXL1 |
| 295 | LOXL2 |
| 296 | LOXL4 |
| 297 | LRRC32 |
| 298 | LSP1 |
| 299 | LTBP2 |
| 300 | LTBR |
| 301 | LTF |
| 302 | LUM |
| 303 | LY96 |
| 304 | LYN |
| 305 | LYZ |
| 306 | LZTS1 |
| 307 | MACC1 |
| 308 | MALL |
| 309 | MAN1C1 |
| 310 | MAP2K3 |
| 311 | MARVELD1 |
| 312 | MCAM |
| 313 | MET |
| 314 | METTL7B |
| 315 | MFAP2 |
| 316 | MGP |
| 317 | MIR155HG |
| 318 | MMP1 |
| 319 | MMP11 |
| 320 | MMP14 |
| 321 | MMP19 |
| 322 | MMP7 |
| 323 | MMP9 |
| 324 | MPZL2 |
| 325 | MPZL3 |
| 326 | MRC2 |
| 327 | MSN |
| 328 | MSR1 |
| 329 | MXRA5 |
| 330 | MXRA7 |
| 331 | MYADM |
| 332 | MYBPH |
| 333 | MYL12A |
| 334 | MYL12B |
| 335 | MYL4 |
| 336 | MYL9 |
| 337 | MYO1G |
| 338 | MYO5B |
| 339 | MYOF |
| 340 | NAMPT |
| 341 | NCF1 |
| 342 | NEK6 |
| 343 | NFKBIZ |
| 344 | NKG7 |
| 345 | NMI |
| 346 | NNMT |
| 347 | NPC2 |
| 348 | NRP1 |
| 349 | NTAN1 |
| 350 | NUAK2 |
| 351 | OAF |
| 352 | OCIAD2 |
| 353 | OLFML2A |
| 354 | OLFML2B |
| 355 | OLFML3 |
| 356 | OR51E1 |
| 357 | OSBPL10 |
| 358 | OSMR |
| 359 | OSTF1 |
| 360 | P2RY6 |
| 361 | P4HA2 |
| 362 | P4HA3 |
| 363 | PCOLCE |
| 364 | PDIA5 |
| 365 | PDK3 |
| 366 | PDLIM1 |
| 367 | PDLIM4 |
| 368 | PDLIM7 |
| 369 | PDPN |
| 370 | PECAM1 |
| 371 | PHLDA2 |
| 372 | PLA2G2A |
| 373 | PLAT |
| 374 | PLAU |
| 375 | PLAUR |
| 376 | PLB1 |
| 377 | PLBD1 |
| 378 | PLEK2 |
| 379 | PLEKHA4 |
| 380 | PLIN2 |
| 381 | PLK3 |
| 382 | PLOD1 |
| 383 | PLOD2 |
| 384 | PLP2 |
| 385 | PLSCR1 |
| 386 | PLXND1 |
| 387 | PODNL1 |
| 388 | POSTN |
| 389 | PPCS |
| 390 | PPM1M |
| 391 | PPP1R15A |
| 392 | PRELID2 |
| 393 | PRF1 |
| 394 | PROCR |
| 395 | PRPS2 |
| 396 | PRR16 |
| 397 | PRSS23 |
| 398 | PTGER2 |
| 399 | PTGER4 |
| 400 | PTGIR |
| 401 | PTPN22 |
| 402 | PTPN7 |
| 403 | PTX3 |
| 404 | PYGL |
| 405 | RAB27A |
| 406 | RAB32 |
| 407 | RAB34 |
| 408 | RAB36 |
| 409 | RAB38 |
| 410 | RAC2 |
| 411 | RARRES1 |
| 412 | RARRES2 |
| 413 | RASSF5 |
| 414 | RBM47 |
| 415 | RBMS1 |
| 416 | RBP1 |
| 417 | RCAN1 |
| 418 | RDH10 |
| 419 | RELB |
| 420 | RETN |
| 421 | REXO2 |
| 422 | RFTN1 |
| 423 | RGS16 |
| 424 | RHOH |
| 425 | RIN1 |
| 426 | RNASE2 |
| 427 | RNASE3 |
| 428 | RNF135 |
| 429 | RNF19B |
| 430 | RRAS |
| 431 | RUNX1 |
| 432 | S100A10 |
| 433 | S100A11 |
| 434 | S100A4 |
| 435 | S100A6 |
| 436 | S100A8 |
| 437 | S100A9 |
| 438 | SAA1 |
| 439 | SAA2 |
| 440 | SAT1 |
| 441 | SDC1 |
| 442 | SEC24D |
| 443 | SECTM1 |
| 444 | SEL1L3 |
| 445 | SEMA3F |
| 446 | SERPINA1 |
| 447 | SERPINA3 |
| 448 | SERPINA5 |
| 449 | SERPINB1 |
| 450 | SERPINB8 |
| 451 | SERPINE1 |
| 452 | SERPINF1 |
| 453 | SERPING1 |
| 454 | SERPINH1 |
| 455 | SERTAD1 |
| 456 | SH2B3 |
| 457 | SH2D2A |
| 458 | SH2D4A |
| 459 | SH3BGRL3 |
| 460 | SHC1 |
| 461 | SHISA5 |
| 462 | SIGLEC9 |
| 463 | SIRPG |
| 464 | SIT1 |
| 465 | SKAP1 |
| 466 | SLC10A3 |
| 467 | SLC11A1 |
| 468 | SLC12A7 |
| 469 | SLC16A3 |
| 470 | SLC25A24 |
| 471 | SLC2A3 |
| 472 | SLC30A7 |
| 473 | SLC43A3 |
| 474 | SLC9A1 |
| 475 | SLN |
| 476 | SMAGP |
| 477 | SNAI1 |
| 478 | SNX10 |
| 479 | SNX20 |
| 480 | SOCS3 |
| 481 | SOD2 |
| 482 | SOD3 |
| 483 | SPAG4 |
| 484 | SPATS2L |
| 485 | SPHK1 |
| 486 | SPINT1 |
| 487 | SPOCD1 |
| 488 | SPON2 |
| 489 | SPP1 |
| 490 | SRPX2 |
| 491 | STC1 |
| 492 | STEAP3 |
| 493 | STK40 |
| 494 | SUSD2 |
| 495 | SWAP70 |
| 496 | SYNPO |
| 497 | TAGLN |
| 498 | TAGLN2 |
| 499 | TBC1D1 |
| 500 | TCEA3 |
| 501 | TDO2 |
| 502 | TEAD3 |
| 503 | TEAD4 |
| 504 | TES |
| 505 | TFPI2 |
| 506 | TGFB2 |
| 507 | TGFBI |
| 508 | THBD |
| 509 | THBS1 |
| 510 | TIFA |
| 511 | TIMP1 |
| 512 | TLR1 |
| 513 | TLR8 |
| 514 | TM4SF1 |
| 515 | TMEM173 |
| 516 | TMEM176B |
| 517 | TMEM61 |
| 518 | TMEM70 |
| 519 | TMEM71 |
| 520 | TMSB10 |
| 521 | TMSB4X |
| 522 | TNFAIP3 |
| 523 | TNFAIP6 |
| 524 | TNFAIP8 |
| 525 | TNFRSF11A |
| 526 | TNFRSF11B |
| 527 | TNFRSF12A |
| 528 | TNFRSF14 |
| 529 | TNFRSF1A |
| 530 | TNFSF14 |
| 531 | TOM1L1 |
| 532 | TPM4 |
| 533 | TPRG1 |
| 534 | TREM1 |
| 535 | TRPV2 |
| 536 | TSPAN2 |
| 537 | TTC39A |
| 538 | TUBB6 |
| 539 | TYMP |
| 540 | UGCG |
| 541 | ULBP2 |
| 542 | UPP1 |
| 543 | VAMP5 |
| 544 | VAMP8 |
| 545 | VASN |
| 546 | VASP |
| 547 | VAV3 |
| 548 | VDR |
| 549 | VIM |
| 550 | VNN2 |
| 551 | WARS |
| 552 | WIPI1 |
| 553 | WWTR1 |
| 554 | YIPF1 |
| 555 | ZDHHC12 |
| 526 | TNFRSF11B |
| 527 | TNFRSF12A |
| 528 | TNFRSF14 |
| 529 | TNFRSF1A |
| 530 | TNFSF14 |
| 531 | TOM1L1 |
| 532 | TPM4 |
| 533 | TPRG1 |
| 534 | TREM1 |
| 535 | TRPV2 |
| 536 | TSPAN2 |
| 537 | TTC39A |
| 538 | TUBB6 |
| 539 | TYMP |
| 540 | UGCG |
| 541 | ULBP2 |
| 542 | UPP1 |
| 543 | VAMP5 |
| 544 | VAMP8 |
| 545 | VASN |
| 546 | VASP |
| 547 | VAV3 |
| 548 | VDR |
| 549 | VIM |
| 550 | VNN2 |
| 551 | WARS |
| 552 | WIPI1 |
| 553 | WWTR1 |
| 554 | YIPF1 |
| 555 | ZDHHC12 |

***BDKRB2-negatively -correlated genes***

| 1 | ATOH8 |
| --- | --- |
| 2 | CDH20 |
| 3 | DLL1 |
| 4 | DLL3 |
| 5 | DSCAML1 |
| 6 | HES6 |
| 7 | HIP1R |
| 8 | IGFN1 |
| 9 | KLF15 |
| 10 | NDRG2 |
| 11 | NEU4 |
| 12 | NOG |
| 13 | PHYHIPL |
| 14 | RASL10A |
| 15 | RFTN2 |
| 16 | SHD |
| 17 | SMOC1 |
| 18 | SOX8 |
| 19 | THRA |
| 20 | ZDHHC22 |
