# Supplementary material for: BDKRB2 is a novel EMT-related biomarker and predicts poor survival in glioma
Source: Aging (Albany NY). 2021 Mar 3;13(5):7499–516. doi: 10.18632/aging.202614 (PMC7993731; doi:10.18632/aging.202614)
Supplement: Supplementary Table 2 [file aging-13-202614-s003.docx]

| \| **Supplementary Table 2. Gene list for BDKRB2-significantly-correlated genes that overlap between CGGA and TCGA GBM.** \| \| --- \| \| \| |
| --- | --- |
|  |
|  |

***BDKRB2-positively-correlated genes***

| 1 | ABCC3 |
| --- | --- |
| 2 | ACTA2 |
| 3 | ACTG2 |
| 4 | ACTN1 |
| 5 | ADAM12 |
| 6 | ADAM8 |
| 7 | ADAMTS1 |
| 8 | ADAMTS14 |
| 9 | ADAP2 |
| 10 | AHNAK2 |
| 11 | ALOX5 |
| 12 | ANGPTL4 |
| 13 | ANO1 |
| 14 | ANPEP |
| 15 | ANXA2 |
| 16 | ANXA2P1 |
| 17 | AQP9 |
| 18 | ARHGDIB |
| 19 | ARPC1B |
| 20 | ATP13A3 |
| 21 | B4GALT1 |
| 22 | BACE2 |
| 23 | BATF |
| 24 | BCL2A1 |
| 25 | BCL3 |
| 26 | BDKRB1 |
| 27 | BDKRB2 |
| 28 | BGN |
| 29 | BHLHE40 |
| 30 | BIRC3 |
| 31 | C1R |
| 32 | C1RL |
| 33 | C1S |
| 34 | C5AR1 |
| 35 | CCL2 |
| 36 | CCL20 |
| 37 | CCL7 |
| 38 | CCR1 |
| 39 | CCR5 |
| 40 | CD14 |
| 41 | CD163 |
| 42 | CD248 |
| 43 | CD40 |
| 44 | CD44 |
| 45 | CD55 |
| 46 | CD93 |
| 47 | CDCP1 |
| 48 | CEBPB |
| 49 | CHI3L1 |
| 50 | CHRDL2 |
| 51 | CLCF1 |
| 52 | CLDN23 |
| 53 | CNN1 |
| 54 | COL13A1 |
| 55 | COL15A1 |
| 56 | COL18A1 |
| 57 | COL1A1 |
| 58 | COL3A1 |
| 59 | COL4A1 |
| 60 | COL5A1 |
| 61 | COL6A2 |
| 62 | COL6A3 |
| 63 | COL8A1 |
| 64 | CTSB |
| 65 | CTSC |
| 66 | CTSZ |
| 67 | CXCL3 |
| 68 | CXCL6 |
| 69 | CYP1B1 |
| 70 | CYTH4 |
| 71 | DCBLD2 |
| 72 | DOK3 |
| 73 | DPYD |
| 74 | DSE |
| 75 | EGFL6 |
| 76 | EHD4 |
| 77 | ELF4 |
| 78 | EMB |
| 79 | ESM1 |
| 80 | F13A1 |
| 81 | FAM114A1 |
| 82 | FAM20A |
| 83 | FAM20C |
| 84 | FBLIM1 |
| 85 | FCER1G |
| 86 | FCGR2A |
| 87 | FCGR2B |
| 88 | FERMT3 |
| 89 | FHOD1 |
| 90 | FN1 |
| 91 | FNDC3B |
| 92 | FOSL1 |
| 93 | FOSL2 |
| 94 | GALNT5 |
| 95 | GCNT1 |
| 96 | GLIPR1 |
| 97 | GNA15 |
| 98 | GPR84 |
| 99 | GPRC5A |
| 100 | HAMP |
| 101 | HK3 |
| 102 | HMOX1 |
| 103 | HRH1 |
| 104 | HSPA6 |
| 105 | IBSP |
| 106 | ICAM1 |
| 107 | IER3 |
| 108 | IGFBP4 |
| 109 | IKBIP |
| 110 | IL11 |
| 111 | IL1B |
| 112 | IL1R1 |
| 113 | IL21R |
| 114 | IL2RA |
| 115 | IL2RB |
| 116 | IL4R |
| 117 | IL6 |
| 118 | ITGA1 |
| 119 | ITGA3 |
| 120 | ITGA5 |
| 121 | ITGB1 |
| 122 | ITGB3 |
| 123 | JAK3 |
| 124 | KCNE3 |
| 125 | KCNK6 |
| 126 | KCNN4 |
| 127 | KYNU |
| 128 | LAIR1 |
| 129 | LAMB1 |
| 130 | LIF |
| 131 | LILRB2 |
| 132 | LILRB3 |
| 133 | LMNA |
| 134 | LOX |
| 135 | LOXL1 |
| 136 | LOXL2 |
| 137 | LRRC25 |
| 138 | LRRC32 |
| 139 | LTBP2 |
| 140 | LY96 |
| 141 | MALL |
| 142 | MAP3K8 |
| 143 | MAPK13 |
| 144 | MARCO |
| 145 | METRNL |
| 146 | MMP1 |
| 147 | MMP11 |
| 148 | MMP19 |
| 149 | MMP7 |
| 150 | MPZL2 |
| 151 | MPZL3 |
| 152 | MVP |
| 153 | MXRA5 |
| 154 | MYADM |
| 155 | MYL12A |
| 156 | MYL9 |
| 157 | MYO1G |
| 158 | MYOF |
| 159 | NAMPT |
| 160 | NDRG1 |
| 161 | NFKBIZ |
| 162 | NOD2 |
| 163 | NRP1 |
| 164 | OAF |
| 165 | OLFML2B |
| 166 | OR51E1 |
| 167 | OSMR |
| 168 | OSTF1 |
| 169 | P2RY6 |
| 170 | P4HA2 |
| 171 | P4HA3 |
| 172 | PDIA5 |
| 173 | PDK3 |
| 174 | PDLIM7 |
| 175 | PHLDA2 |
| 176 | PLAU |
| 177 | PLAUR |
| 178 | PLB1 |
| 179 | PLEK2 |
| 180 | PLK3 |
| 181 | PLP2 |
| 182 | PLVAP |
| 183 | PLXND1 |
| 184 | PODNL1 |
| 185 | POLD4 |
| 186 | PPP1R3B |
| 187 | PRF1 |
| 188 | PRSS23 |
| 189 | PTGIR |
| 190 | PTPN7 |
| 191 | PTX3 |
| 192 | RAB27A |
| 193 | RAB38 |
| 194 | RAC2 |
| 195 | RARRES1 |
| 196 | RBM47 |
| 197 | RDH10 |
| 198 | RELB |
| 199 | RNASE2 |
| 200 | RNASE3 |
| 201 | RUNX1 |
| 202 | RUNX2 |
| 203 | S100A11 |
| 204 | S100A8 |
| 205 | S100A9 |
| 206 | SAT1 |
| 207 | SBNO2 |
| 208 | SERPINA1 |
| 209 | SERPINB1 |
| 210 | SERPINB8 |
| 211 | SERPINE1 |
| 212 | SGMS2 |
| 213 | SH2B3 |
| 214 | SHC1 |
| 215 | SIGLEC9 |
| 216 | SLC16A3 |
| 217 | SLC25A19 |
| 218 | SLC2A3 |
| 219 | SLC39A8 |
| 220 | SLC6A6 |
| 221 | SNX20 |
| 222 | SNX9 |
| 223 | SOCS3 |
| 224 | SOD2 |
| 225 | SPAG4 |
| 226 | SPHK1 |
| 227 | SPOCD1 |
| 228 | SPON2 |
| 229 | SRPX2 |
| 230 | STAB1 |
| 231 | SVIL |
| 232 | TACSTD2 |
| 233 | TAGLN |
| 234 | TDO2 |
| 235 | TES |
| 236 | TGFBI |
| 237 | THBD |
| 238 | THBS1 |
| 239 | TIMP1 |
| 240 | TMEM173 |
| 241 | TNFAIP2 |
| 242 | TNFAIP3 |
| 243 | TNFAIP8 |
| 244 | TNFRSF1A |
| 245 | TNFSF14 |
| 246 | TPM2 |
| 247 | TPM4 |
| 248 | TREM1 |
| 249 | TRPV2 |
| 250 | UPP1 |
| 251 | VASN |
| 252 | VASP |
| 253 | VCL |
| 254 | VDR |
| 255 | VENTX |
| 256 | WWTR1 |
| 257 | ZC3H12A |
