# Supplementary material for: BDKRB2 is a novel EMT-related biomarker and predicts poor survival in glioma
Source: Aging (Albany NY). 2021 Mar 3;13(5):7499–516. doi: 10.18632/aging.202614 (PMC7993731; doi:10.18632/aging.202614)
Supplement: Supplementary Table 3 [file aging-13-202614-s004.docx]

**Supplementary Table 3. GO terms of BDKRB2-significantly-correlated genes.**

| GO terms of BDKRB2-**positively**-correlated genes in **pan-glioma** | | | |
| --- | --- | --- | --- |
| GO Term | Count | *P* value | Genes |
| GO:0030198~extracellular matrix organization | 43 | 1.53E-23 | IBSP, MPZL3, LUM, COL3A1, POSTN, ITGB3, ITGB1, TNFRSF11B, CD44, SERPINE1, TGFBI, COL6A3, COL6A2, BCL3, LOX, LAMB1, COL8A1, THBS1, LOXL1, SPP1, FN1, ICAM1, F11R, COL4A2, COL4A1, EGFL6, OLFML2B, HSPG2, SPINT1, OLFML2A, ITGA3, ITGA4, COL5A2, COL5A1, EMILIN1, BGN, ITGA5, FBLN5, PECAM1, COL1A2, MFAP2, COL1A1, LAMC1 |
| GO:0006954~inflammatory response | 57 | 1.29E-22 | RARRES2, S100A8, TSPAN2, TLR1, S100A9, IL15, IL10, TLR8, CXCL10, PTGIR, TNFRSF11B, CASP4, TNFRSF11A, SERPINA3, FAS, CIITA, NFKBIZ, F11R, PTGER2, C5AR1, LTBR, GBP5, LYN, LY96, RELB, LYZ, TNFRSF14, CD40, GAL, TNFAIP6, SDC1, CCR5, CCR2, TNFAIP3, CCL2, NMI, FPR1, BDKRB1, FPR2, BDKRB2, CXCL6, CCL5, CCL7, SLC11A1, TNFRSF1A, HRH1, CCL20, THBS1, PTX3, SPP1, IL6, IL2RA, SPHK1, CHI3L1, ANXA1, AOX1, CD14 |
| GO:0050900~leukocyte migration | 30 | 6.21E-18 | CAV1, MMP9, FPR1, FPR2, ITGB3, ITGB1, MMP1, CD44, PROCR, CD2, FCER1G, SHC1, MSN, FN1, ICAM1, F11R, C5AR1, LYN, ITGA3, ITGA4, SLC16A3, SIRPG, DOK2, THBD, ITGA5, CD58, PECAM1, COL1A2, COL1A1, TREM1 |
| GO:0006955~immune response | 52 | 6.30E-17 | CD8A, AQP9, TLR1, SUSD2, TNFSF14, IL15, HLA-DMA, IL10, CXCL10, CD96, TNFRSF11B, HAMP, IL4R, FAS, FCGR3A, CIITA, LTBR, C5AR1, PTGER4, GZMA, TNFRSF14, CD40, CCR5, CST7, CCR2, CTSC, GBP2, LCP2, IL1R1, CCL2, HLA-DRB1, IFITM2, IFITM3, GPR65, C1R, CD70, CXCL6, IL7R, CCL5, LIF, SLC11A1, TNFRSF1A, CCL20, HLA-DOA, THBS1, SECTM1, IL6, IL2RA, IL1RN, CD276, FCGR2B, CD274 |
| GO:0030574~collagen catabolic process | 19 | 5.80E-13 | COL4A2, COL4A1, MMP9, COL3A1, MMP19, MRC2, COL15A1, MMP7, MMP14, COL5A2, COL5A1, MMP1, MMP11, COL6A3, COL1A2, COL6A2, COL1A1, CTSB, COL8A1 |
| GO:0032496~response to lipopolysaccharide | 27 | 8.58E-12 | S100A8, PTPN22, BDKRB1, CXCL6, GNG12, CXCL10, TNFRSF1A, CD96, SLC11A1, PTGIR, TNFRSF11B, TNFRSF11A, CASP8, FAS, CD6, CASP1, LOXL1, PTGER2, C5AR1, LTBR, PTGER4, LY96, TNFRSF14, CD40, SOD2, THBD, ADM |
| GO:0001525~angiogenesis | 30 | 7.95E-11 | SAT1, CAV1, CCL2, NRP1, TNFRSF12A, ESM1, ELK3, TGFB2, WARS, TYMP, ANG, HMOX1, TGFBI, SERPINE1, SHC1, COL8A1, PLXND1, FN1, COL4A2, VAV3, MMP19, HSPG2, COL15A1, MMP14, MCAM, ANXA2, SH2D2A, SRPX2, ITGA5, PECAM1 |
| GO:0007155~cell adhesion | 43 | 4.70E-10 | IBSP, MPZL3, CCL2, POSTN, FPR2, ITGB3, SIGLEC9, ISLR, CD96, CD44, TGFBI, MYBPH, COL6A3, CD2, COL6A2, SPON2, LAMB1, LOXL2, COL8A1, THBS1, SPP1, FN1, ICAM1, F11R, EGFL6, EFNB2, COL15A1, ITGA3, ITGA4, MCAM, EMILIN2, COL5A1, EMILIN1, TNFAIP6, SIRPG, ITGA5, CD58, PECAM1, FBLN7, COL1A1, LAMC1, ADAM12, ENG |
| GO:0006935~chemotaxis | 21 | 1.22E-09 | PLP2, RARRES2, CCL2, RNASE2, C5AR1, FPR1, CXCL6, FPR2, FES, CCL5, CCL7, PLAUR, CXCL10, LSP1, TYMP, RAC2, CCR5, CCL20, CCR2, FOSL1, PLAU |
| GO:0030199~collagen fibril organization | 13 | 1.39E-09 | FMOD, LUM, COL3A1, COL5A2, SERPINH1, COL5A1, ANXA2, TGFB2, MMP11, COL1A2, LOX, COL1A1, LOXL2 |
| GO:0002576~platelet degranulation | 19 | 2.86E-09 | RARRES2, LYN, F13A1, ACTN1, SERPING1, HGF, ITGB3, CD63, FLNA, TIMP1, TGFB2, ISLR, PECAM1, SERPINE1, SERPINA3, SERPINA1, TMSB4X, THBS1, FN1 |
| GO:0071260~cellular response to mechanical stimulus | 16 | 3.98E-09 | LTBR, PTGER4, MMP7, TNFSF14, CD40, TLR8, CASP5, TNFRSF1A, CASP8, IRF1, COL1A1, FAS, CASP1, GADD45A, ENG, SLC9A1 |
| GO:0006953~acute-phase response | 12 | 1.91E-08 | PLSCR1, IL6, SAA2, ASS1, SAA1, HAMP, IL1RN, SERPINA3, HFE, SERPINA1, FN1, CD163 |
| GO:0007165~signal transduction | 72 | 3.67E-08 | NAMPT, IMPA2, S100A6, NRP1, IGFBP6, S100A9, TLR1, TNFSF14, IL15, IQGAP1, CXCL10, TNFRSF11B, TNFRSF11A, CHRNA9, IL4R, SHC1, FAS, CASP1, ARHGAP9, C5AR1, SIT1, LTBR, LYN, PDPN, CLIC1, PLAUR, TNFAIP6, SH2D2A, DOK2, THBD, ADM, RIN1, CLEC5A, NEK6, OSTF1, CCL2, FPR1, CD70, CXCL6, ELK3, GNG12, IL7R, GPRC5A, CCL7, TNFRSF1A, VDR, CCL20, RAC2, SH2B3, HLA-DOA, RUNX1, ITK, SECTM1, IL2RB, LGALS1, MAP2K3, MET, MRC2, SPHK1, S100A11, ANXA1, COL15A1, RCAN1, OR51E1, LSP1, TOM1L1, FCGR2B, PECAM1, CD274, IGFBP2, GDF15, PLAU |
| GO:0045087~innate immune response | 37 | 8.45E-08 | S100A8, ELF4, TLR1, S100A9, CAPZA1, APOBEC3G, C1R, C1S, FES, APOBEC3F, TLR8, APOBEC3C, TMEM173, CASP4, ANG, SAA1, FCER1G, CFI, C2, SPON2, CD6, PTX3, ITK, LYN, LGALS3, NCF1, LY96, RELB, ANXA1, SERPING1, APOL1, C1RL, JAK3, TREM1, RNF135, CLEC5A, CD14 |
| GO:0042060~wound healing | 15 | 1.63E-07 | IL6, S100A8, COL3A1, CELSR1, ELK3, ITGB3, TIMP1, TGFB2, SLC11A1, SDC1, CCL20, PECAM1, LOX, ENG, FN1 |
| GO:0009612~response to mechanical stimulus | 13 | 2.26E-07 | CCL2, PTGER4, COL3A1, CHI3L1, BDKRB1, POSTN, RCAN1, BDKRB2, MMP14, RETN, IGFBP2, THBS1, FOSL1 |
| GO:0001666~response to hypoxia | 21 | 4.52E-07 | PLAT, CAV1, LDHA, CCL2, PDLIM1, POSTN, MMP14, SOD3, SOD2, TGFB2, CA9, PLOD1, ADM, ANG, PLOD2, HMOX1, THBS1, LOXL2, CASP1, ENG, PLAU |
| GO:0030168~platelet activation | 17 | 5.72E-07 | IL6, GNA15, VAV3, LYN, PDPN, COL3A1, ACTN1, CD40, ITGB3, FLNA, PLSCR1, RAC2, SAA1, COL1A2, FCER1G, COL1A1, LCP2 |
| GO:0022617~extracellular matrix disassembly | 14 | 5.93E-07 | MMP9, MMP19, MMP7, HSPG2, MMP14, MMP1, MMP11, TIMP1, CD44, CAPG, LAMC1, ENG, SPP1, FN1 |
| GO:0030593~neutrophil chemotaxis | 13 | 8.14E-07 | VAV3, C5AR1, CCL2, S100A8, LGALS3, S100A9, CCL5, CCL7, TGFB2, CCL20, SAA1, FCER1G, TREM1 |
| GO:0007568~aging | 20 | 1.02E-06 | CIITA, IL6, CCL2, ASS1, COL3A1, BCL2A1, MMP7, SERPING1, IL15, GCLM, IL10, TIMP1, RETN, ADM, SERPINF1, HAMP, CASP7, CTSC, IGFBP2, LOXL2 |
| GO:0007166~cell surface receptor signaling pathway | 26 | 1.77E-06 | IL1R1, CCL2, CD8A, TSPAN2, CD247, BDKRB2, IL7R, SIGLEC9, CXCL10, CLCF1, CASP8, CD2, IFNGR2, GPR157, IL2RA, CD3D, LY96, MET, ANXA1, TNFRSF14, BIRC3, DOK2, CCR5, LILRB3, CD274, CD14 |
| GO:0007596~blood coagulation | 20 | 5.22E-06 | PLAT, LYN, F13A1, CAPZA1, ITGB3, PLAUR, THBD, PROCR, F3, SERPINA5, COL1A2, IRF1, SH2B3, FCER1G, SERPINA1, COL1A1, TFPI2, EHD2, PLAU, RAB27A |
| GO:0010951~negative regulation of endopeptidase activity | 16 | 5.59E-06 | CAST, SPINT1, SERPING1, SERPINH1, TIMP1, CARD16, SERPINF1, SERPINB8, SERPINA5, COL6A3, SERPINE1, SERPINA3, SERPINB1, SERPINA1, CSTA, TFPI2 |
| GO:0071222~cellular response to lipopolysaccharide | 15 | 1.15E-05 | ICAM1, IL6, CCL2, ASS1, CD40, IL10, CXCL10, CD80, CCL20, CCR5, HAMP, SERPINE1, TNFAIP3, SPON2, CD14 |
| GO:0035987~endodermal cell differentiation | 8 | 1.45E-05 | COL4A2, ITGA5, MMP9, ITGA4, MMP14, COL8A1, LAMB1, FN1 |
| GO:0042832~defense response to protozoan | 7 | 1.74E-05 | BATF, SLC11A1, IL6, IL4R, BCL3, CD40, IL10 |
| GO:0007204~positive regulation of cytosolic calcium ion concentration | 16 | 1.95E-05 | GNA15, PTGER2, C5AR1, PTGER4, SWAP70, FPR1, CD52, BDKRB1, FPR2, BDKRB2, PTGIR, CCR5, ADM, CHRNA9, SAA1, CCR2 |
| GO:0006968~cellular defense response | 11 | 2.04E-05 | LSP1, PRF1, ITK, C5AR1, CCR5, NCF1, LY96, CCR2, CLEC5A, FOSL1, ITGB1 |
| GO:0045071~negative regulation of viral genome replication | 9 | 2.75E-05 | PLSCR1, IFITM2, IFITM3, LTF, APOBEC3G, CCL5, APOBEC3F, APOBEC3C, ISG20 |
| GO:0060326~cell chemotaxis | 11 | 3.13E-05 | CCL2, C5AR1, SAA2, CCL20, FPR1, CXCL6, FPR2, HGF, CCL5, ENG, CXCL10 |
| GO:0050776~regulation of immune response | 18 | 4.46E-05 | ICAM1, CD8A, CD3D, COL3A1, CD247, CD276, CD40, ITGA4, ITGB1, SIGLEC9, CD96, FCGR2B, CLEC2B, IRF1, COL1A2, COL1A1, TREM1, FCGR3A |
| GO:0051607~defense response to virus | 17 | 6.18E-05 | PRF1, IL6, CD8A, IFITM2, IFITM3, APOBEC3G, CD40, APOBEC3F, TLR8, APOBEC3C, CXCL10, ISG20, PLSCR1, TMEM173, IRF1, SPON2, GBP1 |
| GO:0030335~positive regulation of cell migration | 18 | 6.77E-05 | LYN, SPHK1, HGF, MCAM, CCL5, MMP14, SNAI1, MYADM, CCL7, TNFAIP6, ITGA5, F3, SEMA3F, CD274, COL1A1, THBS1, LAMB1, PLAU |
| GO:0042127~regulation of cell proliferation | 18 | 7.24E-05 | SAT1, ITK, PTGER2, LTBR, ANXA1, S100A11, TNFRSF14, CXCL6, CD40, FES, CXCL10, TNFRSF1A, TNFRSF11B, IL4R, FAS, ENG, PLAU, TES |
| GO:0006928~movement of cell or subcellular component | 12 | 7.38E-05 | LSP1, ARPC1B, ARPC2, VIM, CAPZA1, MSN, FPR2, ARPC5, TPM4, VNN2, ARHGDIB, PLAUR |
| GO:0050729~positive regulation of inflammatory response | 11 | 8.68E-05 | TNFRSF1A, CCL2, S100A8, PTGER4, CCR2, SERPINE1, S100A9, PLA2G2A, IL15, CCL5, CCL7 |
| GO:0042493~response to drug | 24 | 9.02E-05 | ICAM1, IL6, LDHA, VAV3, LYN, ASS1, LGALS1, ANXA1, ITGA3, GAL, GCLM, IL10, TGFB2, SOD2, TNFRSF11B, PLIN2, CA9, COL1A1, LOX, IGFBP2, THBS1, FOSL1, SLC9A1, NNMT |
| GO:0016477~cell migration | 17 | 1.01E-04 | CTHRC1, CD248, BDKRB1, ITGB3, ARPC5, CD63, SNAI1, ITGB1, COL5A1, TGFB2, SDC1, ANG, LAMC1, JAK3, THBS1, ENG, SLC9A1 |
| GO:0006915~apoptotic process | 36 | 1.06E-04 | STEAP3, PRF1, S100A8, NUAK2, S100A9, GPR65, TNFSF14, CASP5, TMEM173, CASP4, CASP7, CASP8, CD2, FAS, CASP1, PHLDA2, LTBR, C5AR1, IL2RA, NCF1, GZMA, LGALS1, CHI3L1, BIRC3, AHR, PLSCR1, RASSF5, PLK3, TNFAIP8, IRF1, CTSC, TNFAIP3, GADD45A, PPP1R15A, CD14, NEK6 |
| GO:0042981~regulation of apoptotic process | 19 | 1.32E-04 | STEAP3, LGALS1, ACTN1, TNFRSF14, BIRC3, CASP5, TNFRSF1A, RASSF5, TNFRSF11B, TNFRSF11A, CASP4, CARD16, CASP8, BCL3, CTSB, FAS, CASP1, GDF15, DEDD2 |
| GO:0007267~cell-cell signaling | 21 | 1.50E-04 | NAMPT, NRP1, EFNB2, S100A9, CD70, CXCL6, IL15, CCL5, IL10, CCL7, CXCL10, TGFB2, TNFAIP6, PTGIR, SIRPG, TNFRSF11A, CCR5, CCL20, ADM, CD80, GDF15 |
| GO:0031295~T cell costimulation | 11 | 1.53E-04 | CAV1, CD3D, HLA-DRB1, LYN, CD80, LGALS1, CD274, EFNB2, CD247, TNFSF14, TNFRSF14 |
| GO:0071356~cellular response to tumor necrosis factor | 13 | 1.67E-04 | ICAM1, IL6, CCL2, ASS1, CHI3L1, POSTN, CCL5, CCL7, CCL20, HAMP, CD58, COL1A1, THBS1 |
| GO:2000406~positive regulation of T cell migration | 5 | 1.71E-04 | CCL20, TNFRSF14, ITGA4, CCL5, CXCL10 |
| GO:0043627~response to estrogen | 10 | 1.79E-04 | ARPC1B, LDHA, CAV1, TNFRSF11B, IL4R, HMOX1, RCAN1, IGFBP2, MMP14, GAL |
| GO:0001503~ossification | 11 | 1.89E-04 | IBSP, OSTF1, TNFRSF11A, PDLIM7, MMP9, LTF, MGP, STC1, RUNX1, COL5A2, SPP1 |
| GO:0046718~viral entry into host cell | 11 | 1.89E-04 | ICAM1, F11R, CD80, VAMP8, ITGA5, EFNB2, TNFRSF14, CTSB, ITGB3, CLEC5A, ITGB1 |
| GO:0050731~positive regulation of peptidyl-tyrosine phosphorylation | 11 | 2.33E-04 | LIF, ICAM1, IL6, NRP1, CD44, CD80, ITGA5, TNFRSF14, HGF, ITGB3, EHD4 |
| GO:0010759~positive regulation of macrophage chemotaxis | 5 | 2.62E-04 | RARRES2, CCL2, C5AR1, THBS1, CCL5 |
| GO:0002548~monocyte chemotaxis | 8 | 2.93E-04 | IL6, TNFRSF11A, CCL2, LGALS3, CCL20, ANXA1, CCL5, CCL7 |
| GO:0060333~interferon-gamma-mediated signaling pathway | 10 | 3.54E-04 | CIITA, ICAM1, NMI, CD44, HLA-DRB1, IRF1, IFI30, IFNGR2, GBP2, GBP1 |
| GO:0042730~fibrinolysis | 6 | 3.94E-04 | PLAT, SERPINE1, SERPING1, PLAU, ANXA2, PLAUR |
| GO:0031663~lipopolysaccharide-mediated signaling pathway | 7 | 4.11E-04 | CCL2, LYN, LY96, PTPN22, CCL5, CD6, CD14 |
| GO:0008360~regulation of cell shape | 14 | 4.52E-04 | ICAM1, IL6, CCL2, PDPN, HEXB, ANXA1, ARHGAP18, FBLIM1, MYL12B, MYL12A, MSN, FES, CCL7, FN1 |
| GO:1902042~negative regulation of extrinsic apoptotic signaling pathway via death domain receptors | 7 | 4.90E-04 | ICAM1, HMOX1, CASP8, SERPINE1, FAS, HGF, TNFAIP3 |
| GO:0034113~heterotypic cell-cell adhesion | 6 | 4.96E-04 | ITGA5, CD58, CD2, ITGA4, ITGB3, ITGB1 |
| GO:0050918~positive chemotaxis | 7 | 6.81E-04 | NRP1, LGALS3, SAA2, SAA1, MET, HGF, CCL5 |
| GO:0035729~cellular response to hepatocyte growth factor stimulus | 5 | 7.36E-04 | IL6, NRP1, HGF, GCLM, IL10 |
| GO:0050728~negative regulation of inflammatory response | 10 | 7.85E-04 | TNFRSF1A, TNFAIP6, IL2RA, PTGER4, SERPINF1, SOCS3, SAA1, CD276, HGF, TNFAIP3 |
| GO:0007159~leukocyte cell-cell adhesion | 6 | 9.27E-04 | ICAM1, ITGA5, MSN, ITGA4, CCL5, ITGB1 |
| GO:0045766~positive regulation of angiogenesis | 12 | 9.53E-04 | C5AR1, ADM, F3, HMOX1, SERPINE1, SPHK1, CHI3L1, RRAS, HGF, THBS1, RUNX1, ENG |
| GO:0007229~integrin-mediated signaling pathway | 11 | 0.001056337 | IBSP, VAV3, ITGA5, COL3A1, FCER1G, ADAMTS1, ITGA3, ITGA4, ITGB3, ADAM12, ITGB1 |
| GO:0006508~proteolysis | 30 | 0.001104221 | MMP9, MMP7, C1R, C1S, PCOLCE, MMP1, CASP5, CASP4, CASP7, CASP8, LTF, C2, CFI, HTRA3, CASP1, PLAT, CTSZ, CFB, MMP19, HGF, MMP14, MMP11, BACE2, C1RL, ADAMTS1, CTSC, CTSB, PRSS23, ADAM12, PLAU |
| GO:0032570~response to progesterone | 7 | 0.001231183 | PTGER2, CAV1, CCL2, CATSPER1, THBS1, FOSL1, TGFB2 |
| GO:0016337~single organismal cell-cell adhesion | 11 | 0.001232514 | ICAM1, MPZL2, SRPX2, CD44, CD93, PDPN, CD58, CD2, FBLIM1, SHC1, CSTA |
| GO:0090026~positive regulation of monocyte chemotaxis | 5 | 0.001274122 | CCL2, CCR2, SERPINE1, CCL5, CXCL10 |
| GO:0014909~smooth muscle cell migration | 4 | 0.001513128 | PLAT, ARPC5, ITGB3, PLAU |
| GO:0019882~antigen processing and presentation | 8 | 0.001552186 | RAB32, HLA-DRB1, CD8A, RAB34, RELB, ULBP2, HFE, RAB27A |
| GO:0097190~apoptotic signaling pathway | 9 | 0.001633871 | CAV1, TNFRSF11B, TNFRSF11A, LY96, CASP8, TNFRSF14, FAS, CD40, CD14 |
| GO:0043123~positive regulation of I-kappaB kinase/NF-kappaB signaling | 14 | 0.001662845 | S100A4, SECTM1, LTBR, LGALS1, CD40, BIRC3, FLNA, TNFRSF1A, HMOX1, SHISA5, CASP8, LTF, CASP1, NEK6 |
| GO:0071346~cellular response to interferon-gamma | 8 | 0.001916827 | CIITA, CCL2, GBP5, ASS1, CCL20, CD58, CCL5, CCL7 |
| GO:0055072~iron ion homeostasis | 6 | 0.002186165 | STEAP3, SLC11A1, HMOX1, HFE, FTL, SOD2 |
| GO:0044344~cellular response to fibroblast growth factor stimulus | 6 | 0.002186165 | CCL2, CD44, POSTN, COL1A1, CCL5, GCLM |
| GO:0060670~branching involved in labyrinthine layer morphogenesis | 4 | 0.002217179 | ADM, SOCS3, SPINT1, IL10 |
| GO:0048245~eosinophil chemotaxis | 4 | 0.002217179 | HRH1, LGALS3, CCL5, CCL7 |
| GO:0009615~response to virus | 11 | 0.002346003 | BATF3, NPC2, ACTA2, IFITM2, IFITM3, BCL3, CCL5, IFNGR2, FOSL1, TLR8, ISG20 |
| GO:0043066~negative regulation of apoptotic process | 27 | 0.002379557 | NUAK2, MMP9, FHL2, IL10, TIMP1, CD44, STK40, LTF, BCL3, FAS, THBS1, IL2RB, IL6, SOCS3, SPHK1, BCL2A1, ANXA1, BIRC3, FLNA, SOD2, PLAUR, ATF5, AMIGO2, PLK3, TNFAIP8, CLEC5A, SLC9A1 |
| GO:0006874~cellular calcium ion homeostasis | 10 | 0.002498395 | VDR, CAV1, CCL2, ATP2A3, CCR2, HEXB, STC1, CD40, CCL5, CCL7 |
| GO:0042102~positive regulation of T cell proliferation | 8 | 0.002584191 | IL6, CD274, ANXA1, CD276, IL15, JAK3, CCL5, CD6 |
| GO:0045669~positive regulation of osteoblast differentiation | 8 | 0.002584191 | CTHRC1, IL6, PDLIM7, FAM20C, CD276, LTF, CLIC1, HGF |
| GO:0007249~I-kappaB kinase/NF-kappaB signaling | 8 | 0.002584191 | TNFRSF1A, LY96, RELB, BCL3, TIFA, BIRC3, CD14, TLR8 |
| GO:0019221~cytokine-mediated signaling pathway | 12 | 0.002722421 | ASPN, PLP2, TNFRSF1A, IL2RB, IL6, BGN, PODNL1, CCL2, SOCS3, CLCF1, F3, CCR2 |
| GO:0046697~decidualization | 5 | 0.003071753 | LIF, VDR, STC1, CTSB, SPP1 |
| GO:0014911~positive regulation of smooth muscle cell migration | 5 | 0.003071753 | RETN, P2RY6, NRP1, POSTN, CCL5 |
| GO:0042110~T cell activation | 7 | 0.00328335 | ITK, CD8A, CD80, CASP8, CD2, CD276, TNFSF14 |
| GO:0050727~regulation of inflammatory response | 8 | 0.003417103 | CASP5, IL1R1, CASP4, LYN, ANXA1, BIRC3, TNFAIP3, CASP1 |
| GO:0010628~positive regulation of gene expression | 18 | 0.003733849 | IL6, CAV1, ACTA2, LRRC32, VIM, HFE, PTPN22, ITGA3, IL7R, TGFB2, SLC11A1, PLSCR1, VDR, ACTG2, MSN, ENG, FN1, RAB27A |
| GO:0033209~tumor necrosis factor-mediated signaling pathway | 11 | 0.003910395 | TNFRSF1A, TNFRSF11B, TNFRSF11A, LTBR, TNFRSF12A, TNFSF14, TNFRSF14, CD70, FAS, CD40, BIRC3 |
| GO:0032868~response to insulin | 8 | 0.004828079 | RETN, IL6, ADM, LYN, IGFBP2, GAL, GCNT1, IL10 |
| GO:0034329~cell junction assembly | 4 | 0.005414329 | FBLIM1, FLNC, FLNA, VASP |
| GO:0050777~negative regulation of immune response | 4 | 0.005414329 | IL2RA, LYN, COL3A1, TGFB2 |
| GO:0048013~ephrin receptor signaling pathway | 9 | 0.005431145 | ARPC1B, VAV3, LYN, ARPC2, AP2S1, MMP9, EFNB2, MYL12A, ARPC5 |
| GO:0034097~response to cytokine | 7 | 0.005461524 | TNFRSF11A, OSMR, CD274, COL3A1, RELB, FOSL1, TIMP1 |
| GO:0034605~cellular response to heat | 6 | 0.005590538 | LYN, HMOX1, HSPA6, THBS1, MYOF, CXCL10 |
| GO:0032119~sequestering of zinc ion | 3 | 0.005613834 | S100A8, S100A9, SLC30A7 |
| GO:0070232~regulation of T cell apoptotic process | 3 | 0.005613834 | LGALS3, CD274, JAK3 |
| GO:0010574~regulation of vascular endothelial growth factor production | 3 | 0.005613834 | IL6, CCL2, CCR2 |
| GO:0034341~response to interferon-gamma | 5 | 0.006103942 | CIITA, SLC11A1, KYNU, IFITM2, IFITM3 |
| GO:0008284~positive regulation of cell proliferation | 26 | 0.006335918 | NAMPT, OSMR, CD248, ESM1, IL15, ITGB1, CXCL10, TGFB2, TIMP1, LIF, TNFRSF11A, RAC2, CLCF1, SHC1, THBS1, FOSL1, SERTAD1, FN1, IL6, LYN, EFNB2, HGF, WWTR1, SIRPG, ATF3, ADM |
| GO:0070098~chemokine-mediated signaling pathway | 8 | 0.006638361 | CCL2, CCR5, CCL20, CCR2, CXCL6, CCL5, CCL7, CXCL10 |
| GO:0045088~regulation of innate immune response | 4 | 0.006876827 | IRF1, PTPN22, RNF135, BIRC3 |
| GO:0001878~response to yeast | 4 | 0.006876827 | IL6, ADM, ANG, PTX3 |
| GO:0001817~regulation of cytokine production | 4 | 0.006876827 | F11R, LYN, LTF, IQGAP1 |
| GO:0048246~macrophage chemotaxis | 4 | 0.006876827 | CCL2, LGALS3, SAA1, CCL5 |
| GO:0010952~positive regulation of peptidase activity | 4 | 0.006876827 | CAV1, MMP14, PCOLCE, FN1 |
| GO:0050715~positive regulation of cytokine secretion | 5 | 0.007090047 | PTGER4, SAA1, CLEC5A, IL10, CD14 |
| GO:0002040~sprouting angiogenesis | 5 | 0.007090047 | NRP1, ESM1, THBS1, LOXL2, ENG |
| GO:0007160~cell-matrix adhesion | 9 | 0.007124145 | CD96, CD44, FBLN5, COL3A1, ITGA3, ITGA4, ITGB3, CD63, ITGB1 |
| GO:0016049~cell growth | 7 | 0.007846854 | IL6, ITGB3, EMP3, IL7R, EMP1, SLC9A1, TGFB2 |
| GO:0007179~transforming growth factor beta receptor signaling pathway | 9 | 0.008106809 | F11R, CCL2, LTBP2, COL3A1, COL1A2, GDF15, ENG, ITGB1, TGFB2 |
| GO:2001238~positive regulation of extrinsic apoptotic signaling pathway | 5 | 0.008175802 | CAV1, LTBR, TNFRSF12A, G0S2, DEDD2 |
| GO:0070527~platelet aggregation | 6 | 0.008680587 | ACTN1, CLIC1, MYL12A, ITGB3, FLNA, MYL9 |
| GO:0097191~extrinsic apoptotic signaling pathway | 6 | 0.009606453 | TNFRSF12A, CASP8, G0S2, CD70, FAS, TGFB2 |
| GO:0009725~response to hormone | 6 | 0.009606453 | LYN, ANG, MMP19, FHL2, MMP14, TIMP1 |
| GO:0071407~cellular response to organic cyclic compound | 7 | 0.010071292 | TMEM173, P2RY6, MSR1, CCL2, LGALS1, CASP8, CCL5 |
| GO:0033627~cell adhesion mediated by integrin | 4 | 0.010444421 | ICAM1, ITGA5, ITGB3, ITGB1 |
| GO:0001501~skeletal system development | 11 | 0.010925044 | VDR, TNFRSF11B, MMP9, TEAD4, COL3A1, HEXB, COL1A2, POSTN, COL1A1, COL5A2, TGFB2 |
| GO:0098609~cell-cell adhesion | 17 | 0.011477532 | VASN, CAST, F11R, LDHA, SWAP70, CAPZA1, S100A11, ARHGAP18, PDLIM1, TAGLN2, GPRC5A, IQGAP1, VASP, ANXA2, CAPG, TES, EHD4 |
| GO:0006879~cellular iron ion homeostasis | 6 | 0.011657323 | SLC11A1, HAMP, HMOX1, HFE, CP, FTL |
| GO:0048662~negative regulation of smooth muscle cell proliferation | 5 | 0.012068095 | PTGIR, ANG, HMOX1, IL15, TNFAIP3 |
| GO:0090280~positive regulation of calcium ion import | 4 | 0.012560644 | CCL2, LGALS3, TRPV2, STC1 |
| GO:0014002~astrocyte development | 4 | 0.012560644 | S100A8, TSPAN2, VIM, S100A9 |
| GO:0008015~blood circulation | 6 | 0.012786388 | ADM, SERPING1, RCAN1, BDKRB2, MYOF, CXCL10 |
| GO:0007044~cell-substrate junction assembly | 3 | 0.013461476 | ITGA5, ITGB3, FN1 |
| GO:0048525~negative regulation of viral process | 3 | 0.013461476 | LTF, APOBEC3G, APOBEC3F |
| GO:0043615~astrocyte cell migration | 3 | 0.013461476 | CCL2, HEXB, MMP14 |
| GO:0001765~membrane raft assembly | 3 | 0.013461476 | S100A10, RFTN1, ANXA2 |
| GO:0032964~collagen biosynthetic process | 3 | 0.013461476 | COL1A1, SERPINH1, COL5A1 |
| GO:0030449~regulation of complement activation | 5 | 0.013588392 | C5AR1, CFB, CFH, CFI, C2 |
| GO:0010803~regulation of tumor necrosis factor-mediated signaling pathway | 5 | 0.013588392 | TNFRSF1A, CASP8, SPHK1, BIRC3, TNFAIP3 |
| GO:0009611~response to wounding | 7 | 0.013689027 | NRP1, CCL2, ADM, CCR2, FABP5, FN1, TGFB2 |
| GO:0051591~response to cAMP | 6 | 0.013987128 | LDHA, SDC1, THBD, MMP19, COL1A1, FOSL1 |
| GO:1901741~positive regulation of myoblast fusion | 4 | 0.01490393 | IL4R, TNFSF14, GDF15, EHD2 |
| GO:0048535~lymph node development | 4 | 0.01490393 | TNFRSF11A, CD248, IL15, IL7R |
| GO:0031589~cell-substrate adhesion | 4 | 0.01490393 | ITGA5, MYO1G, ITGB3, ITGB1 |
| GO:0045765~regulation of angiogenesis | 5 | 0.015225106 | WARS, IL6, TNFRSF12A, HMOX1, PLXND1 |
| GO:0071230~cellular response to amino acid stimulus | 6 | 0.015261387 | COL4A1, ASS1, COL3A1, COL1A2, COL1A1, COL5A2 |
| GO:0031100~organ regeneration | 6 | 0.015261387 | CCL2, C5AR1, ADM, HGF, PRPS2, NNMT |
| GO:0032760~positive regulation of tumor necrosis factor production | 6 | 0.015261387 | CCL2, LY96, CD2, FCER1G, SPON2, CD14 |
| GO:0042742~defense response to bacterium | 11 | 0.015787141 | SLC11A1, TNFRSF1A, S100A8, CCL20, HAMP, TLR1, S100A9, BCL3, FCER1G, CXCL6, IL10 |
| GO:0051384~response to glucocorticoid | 7 | 0.015800925 | IL6, SDC1, ADM, IL1RN, FAS, IGFBP2, IL10 |
| GO:0007259~JAK-STAT cascade | 5 | 0.016980855 | NMI, CCL2, SOCS3, CLCF1, CCR2 |
| GO:0050830~defense response to Gram-positive bacterium | 8 | 0.017014014 | IL6, C5AR1, ADM, RNASE3, ANG, PLA2G2A, LYZ, TNFRSF14 |
| GO:0045089~positive regulation of innate immune response | 4 | 0.017477013 | PLSCR1, GBP5, CCL5, TLR8 |
| GO:0014070~response to organic cyclic compound | 6 | 0.018037438 | ICAM1, NAMPT, PLIN2, LYN, LUM, MMP14 |
| GO:0019441~tryptophan catabolic process to kynurenine | 3 | 0.01845881 | KYNU, TDO2, KMO |
| GO:0006032~chitin catabolic process | 3 | 0.01845881 | CHI3L1, CHI3L2, CTBS |
| GO:0071526~semaphorin-plexin signaling pathway | 5 | 0.018858009 | NRP1, SEMA3F, MET, PLXND1, FLNA |
| GO:0018149~peptide cross-linking | 6 | 0.019542504 | F13A1, COL3A1, ANXA1, CSTA, THBS1, FN1 |
| GO:0043011~myeloid dendritic cell differentiation | 4 | 0.020281649 | BATF, BATF3, LTBR, RELB |
| GO:0001837~epithelial to mesenchymal transition | 5 | 0.020858686 | S100A4, HGF, LOXL2, SNAI1, TGFB2 |
| GO:0042542~response to hydrogen peroxide | 6 | 0.021127634 | LDHA, SDC1, HMOX1, COL1A1, FOSL1, SOD2 |
| GO:0070374~positive regulation of ERK1 and ERK2 cascade | 12 | 0.021851762 | ICAM1, IL6, NRP1, CCL2, C5AR1, CD44, CCL20, PLA2G2A, CHI3L1, PTPN22, CCL5, CCL7 |
| GO:0071347~cellular response to interleukin-1 | 7 | 0.02346404 | ICAM1, IL6, CCL2, CCL20, CHI3L1, CCL5, CCL7 |
| GO:0034115~negative regulation of heterotypic cell-cell adhesion | 3 | 0.02410737 | IL1RN, MYADM, IL10 |
| GO:1904706~negative regulation of vascular smooth muscle cell proliferation | 3 | 0.02410737 | HMOX1, CNN1, IL10 |
| GO:0010529~negative regulation of transposition | 3 | 0.02410737 | APOBEC3G, APOBEC3F, APOBEC3C |
| GO:0045580~regulation of T cell differentiation | 3 | 0.02410737 | CD2, IL15, HLA-DOA |
| GO:0001766~membrane raft polarization | 3 | 0.02410737 | MALL, MARVELD1, CD2 |
| GO:0043304~regulation of mast cell degranulation | 3 | 0.02410737 | RAC2, LYN, FES |
| GO:0045576~mast cell activation | 3 | 0.02410737 | FCER1G, LCP2, RHOH |
| GO:0019886~antigen processing and presentation of exogenous peptide antigen via MHC class II | 8 | 0.02512439 | AP1S3, HLA-DRB1, AP2S1, IFI30, FCER1G, HLA-DOA, HLA-DMA, SEC24D |
| GO:0010043~response to zinc ion | 5 | 0.025237848 | S100A8, ASS1, HAMP, SLC30A7, SOD2 |
| GO:0009409~response to cold | 5 | 0.025237848 | IL6, ADM, CASP8, SOD2, CXCL10 |
| GO:0019835~cytolysis | 4 | 0.026588154 | PRF1, APOL1, GZMA, LYZ |
| GO:0050829~defense response to Gram-negative bacterium | 6 | 0.028295673 | SLC11A1, IL6, ADM, SERPINE1, LYZ, TNFRSF14 |
| GO:0002230~positive regulation of defense response to virus by host | 4 | 0.030089298 | TMEM173, PTPN22, APOBEC3G, APOBEC3F |
| GO:0001568~blood vessel development | 5 | 0.030130377 | SPHK1, COL1A2, COL1A1, AHR, COL5A1 |
| GO:0034446~substrate adhesion-dependent cell spreading | 5 | 0.030130377 | ITGA4, LAMC1, ITGB3, LAMB1, FN1 |
| GO:0010811~positive regulation of cell-substrate adhesion | 5 | 0.030130377 | EGFL6, ITGA5, ITGA3, COL8A1, EMILIN1 |
| GO:0042517~positive regulation of tyrosine phosphorylation of Stat3 protein | 5 | 0.030130377 | LIF, IL6, SOCS3, CLCF1, IL15 |
| GO:0035456~response to interferon-beta | 3 | 0.030361711 | PLSCR1, IFITM2, IFITM3 |
| GO:0021785~branchiomotor neuron axon guidance | 3 | 0.030361711 | NRP1, SEMA3F, PLXND1 |
| GO:0006569~tryptophan catabolic process | 3 | 0.030361711 | KYNU, TDO2, KMO |
| GO:0060586~multicellular organismal iron ion homeostasis | 3 | 0.030361711 | SLC11A1, HAMP, HFE |
| GO:0048012~hepatocyte growth factor receptor signaling pathway | 3 | 0.030361711 | NRP1, MET, HGF |
| GO:0009972~cytidine deamination | 3 | 0.030361711 | APOBEC3G, APOBEC3F, APOBEC3C |
| GO:0006816~calcium ion transport | 7 | 0.031494929 | VDR, KCNN4, CAV1, SLN, CCR5, ATP2A3, CCL5 |
| GO:0044267~cellular protein metabolic process | 9 | 0.031826151 | SAA1, TGFBI, HEXB, IGFBP6, LYZ, HSPG2, LTF, IGFBP2, MMP1 |
| GO:0055114~oxidation-reduction process | 28 | 0.03270352 | STEAP3, C15ORF48, LDHA, IFI30, IL4I1, PDIA5, KMO, TDO2, P4HA2, PLOD1, PLOD2, P4HA3, GPX8, LOXL4, LOX, LOXL2, LOXL1, GLRX, NCF1, CYB561, SOD3, SOD2, RDH10, AOX1, ALOX5, SH3BGRL3, CP, DPYD |
| GO:0008219~cell death | 5 | 0.032771866 | FOSL2, HMOX1, EMP3, EMP1, TGFB2 |
| GO:0045454~cell redox homeostasis | 7 | 0.033291227 | SLC11A1, IL6, NCF1, LTF, PDIA5, SH3BGRL3, GLRX |
| GO:0009617~response to bacterium | 4 | 0.033820678 | SLC11A1, CAV1, CCL2, FUCA2 |
| GO:0046426~negative regulation of JAK-STAT cascade | 5 | 0.035544487 | ASPN, CAV1, BGN, PODNL1, SOCS3 |
| GO:0006469~negative regulation of protein kinase activity | 8 | 0.035551198 | ASPN, IL6, BGN, PODNL1, SOCS3, GMFG, WWTR1, GADD45A |
| GO:0042116~macrophage activation | 3 | 0.03717858 | SLC11A1, CD93, TLR1 |
| GO:0060674~placenta blood vessel development | 3 | 0.03717858 | SOCS3, SPINT1, FOSL1 |
| GO:0051918~negative regulation of fibrinolysis | 3 | 0.03717858 | THBD, SERPINE1, THBS1 |
| GO:0035646~endosome to melanosome transport | 3 | 0.03717858 | RAB32, RAB38, CD63 |
| GO:0002675~positive regulation of acute inflammatory response | 3 | 0.03717858 | IL6, OSMR, ALOX5AP |
| GO:0019732~antifungal humoral response | 3 | 0.03717858 | ADM, ANG, LTF |
| GO:0010820~positive regulation of T cell chemotaxis | 3 | 0.03717858 | CCR2, TNFSF14, CCL5 |
| GO:0048870~cell motility | 4 | 0.037780211 | SRPX2, PDPN, MMP14, ENG |
| GO:0048661~positive regulation of smooth muscle cell proliferation | 6 | 0.039198851 | RETN, NAMPT, IL6, HMOX1, THBS1, CCL5 |
| GO:0001523~retinoid metabolic process | 6 | 0.041646733 | RDH10, RARRES2, SDC1, PLB1, RBP1, HSPG2 |
| GO:0001816~cytokine production | 4 | 0.041965234 | BATF, ITK, S100A8, S100A9 |
| GO:0034612~response to tumor necrosis factor | 4 | 0.041965234 | TNFRSF11A, CASP8, CHI3L1, CD14 |
| GO:0002250~adaptive immune response | 10 | 0.042994368 | ITK, SIT1, TNFRSF11A, LYN, LILRB3, ANXA1, JAK3, RNF19B, CD6, SKAP1 |
| GO:0008285~negative regulation of cell proliferation | 20 | 0.043369894 | IL6, RARRES1, LYN, IGFBP6, S100A11, IL10, SOD2, TGFB2, LIF, ATF5, WARS, VDR, RASSF5, SIRPG, ADM, IRF1, ADAMTS1, EMP3, FOSL1, TES |
| GO:0016525~negative regulation of angiogenesis | 6 | 0.044184978 | COL4A2, CCL2, SERPINF1, CCR2, THBS1, CXCL10 |
| GO:0042511~positive regulation of tyrosine phosphorylation of Stat1 protein | 3 | 0.044516832 | LIF, TNFRSF1A, CD40 |
| GO:0046135~pyrimidine nucleoside catabolic process | 3 | 0.044516832 | TYMP, UPP1, DPYD |
| GO:0048333~mesodermal cell differentiation | 3 | 0.044516832 | ITGA3, ITGB3, ITGB1 |
| GO:0050707~regulation of cytokine secretion | 3 | 0.044516832 | LYN, TLR1, TLR8 |
| GO:0001886~endothelial cell morphogenesis | 3 | 0.044516832 | PECAM1, MET, STC1 |
| GO:0042535~positive regulation of tumor necrosis factor biosynthetic process | 3 | 0.044516832 | CCR2, TLR1, THBS1 |
| GO:0016064~immunoglobulin mediated immune response | 3 | 0.044516832 | IL4R, FCER1G, TLR8 |
| GO:0002523~leukocyte migration involved in inflammatory response | 3 | 0.044516832 | CCL2, S100A8, S100A9 |
| GO:0001649~osteoblast differentiation | 8 | 0.044534892 | IBSP, MRC2, FHL2, COL1A1, WWTR1, SNAI1, TPM4, SPP1 |
| GO:0045429~positive regulation of nitric oxide biosynthetic process | 5 | 0.044652584 | ICAM1, IL6, ASS1, PTX3, SOD2 |
| GO:0038096~Fc-gamma receptor signaling pathway involved in phagocytosis | 9 | 0.045965943 | ARPC1B, VAV3, LYN, ARPC2, CD247, MYO1G, FCGR2A, ARPC5, FCGR3A |
| GO:0007202~activation of phospholipase C activity | 4 | 0.046372551 | ITK, GNA15, C5AR1, ANG |
| GO:0030512~negative regulation of transforming growth factor beta receptor signaling pathway | 6 | 0.049533877 | ASPN, VASN, CAV2, CAV1, HTRA3, ENG |

| GO terms of BDKRB2-**negatively**-correlated genes in **pan-glioma** | | | |
| --- | --- | --- | --- |
| GO Term | Count | *P* value | Genes |
| GO:0030154~cell differentiation | 5 | 0.001012 | SMOC1, DLL1, NDRG2, HES6, ATOH8 |
| GO:0007386~compartment pattern specification | 2 | 0.004044 | DLL3, DLL1 |
| GO:0045944~positive regulation of transcription from RNA polymerase II promoter | 5 | 0.014948 | NOG, THRA, DLL1, KLF15, SOX8 |
| GO:0001709~cell fate determination | 2 | 0.018076 | DSCAML1, DLL1 |
| GO:0090190~positive regulation of branching involved in ureteric bud morphogenesis | 2 | 0.019071 | NOG, SOX8 |
| GO:0072583~clathrin-mediated endocytosis | 2 | 0.020065 | HIP1R, DLL1 |
| GO:0060173~limb development | 2 | 0.023042 | NOG, SMOC1 |
| GO:0045662~negative regulation of myoblast differentiation | 2 | 0.023042 | DLL1, SOX8 |
| GO:0021510~spinal cord development | 2 | 0.029955 | NOG, DLL1 |
| GO:0009953~dorsal/ventral pattern formation | 2 | 0.031922 | NOG, DSCAML1 |
| GO:0007399~nervous system development | 3 | 0.033435 | NOG, HES6, ATOH8 |
| GO:0001756~somitogenesis | 2 | 0.038776 | DLL3, DLL1 |

| GO terms of BDKRB2-**positively**-correlated genes in **glioblastoma (GBM)** | | | |
| --- | --- | --- | --- |
| GO Term | Count | *P* value | Genes |
| GO:0030198~extracellular matrix organization | 30 | 6.39E-21 | IBSP, MPZL3, COL3A1, ITGB3, ITGB1, CD44, TGFBI, COL6A3, SERPINE1, COL6A2, BCL3, LOX, COL8A1, THBS1, LAMB1, LOXL1, FN1, COL18A1, B4GALT1, ICAM1, COL4A1, COL13A1, EGFL6, OLFML2B, ITGA1, ITGA3, COL5A1, BGN, ITGA5, COL1A1 |
| GO:0006954~inflammatory response | 33 | 1.37E-15 | CCL2, S100A8, CCR1, CXCL3, S100A9, BDKRB1, BDKRB2, CXCL6, CCL7, TNFRSF1A, HRH1, PTGIR, CCL20, IL1B, ZC3H12A, PTX3, THBS1, ADAM8, NFKBIZ, IL6, C5AR1, CEBPB, IL2RA, LY96, RELB, SPHK1, CHI3L1, CD40, CCR5, STAB1, TNFAIP3, CD14, IGFBP4 |
| GO:0030574~collagen catabolic process | 16 | 1.90E-14 | COL18A1, COL4A1, ADAMTS14, COL13A1, COL3A1, MMP19, COL15A1, MMP7, COL5A1, MMP1, MMP11, COL6A3, COL6A2, COL1A1, CTSB, COL8A1 |
| GO:0001525~angiogenesis | 23 | 2.12E-12 | COL18A1, SAT1, NRP1, CCL2, CYP1B1, MMP19, COL15A1, ANPEP, ESM1, ANXA2, SRPX2, ITGA5, HMOX1, TGFBI, SERPINE1, ZC3H12A, SHC1, COL8A1, PLXND1, ADAM8, TNFAIP2, FN1, ANGPTL4 |
| GO:0007155~cell adhesion | 29 | 2.18E-10 | IBSP, MPZL3, CCL2, CYP1B1, CCR1, ITGB3, SIGLEC9, VCL, CD44, TGFBI, COL6A3, COL6A2, LOXL2, COL8A1, THBS1, LAMB1, SPON2, FN1, COL18A1, B4GALT1, ICAM1, EGFL6, COL15A1, ITGA3, COL5A1, ITGA5, STAB1, COL1A1, ADAM12 |
| GO:0050900~leukocyte migration | 16 | 3.43E-10 | B4GALT1, ICAM1, C5AR1, ITGA3, ITGB3, ITGB1, MMP1, SLC16A3, THBD, CD44, ITGA5, FCER1G, SHC1, TREM1, COL1A1, FN1 |
| GO:0071222~cellular response to lipopolysaccharide | 15 | 1.22E-09 | ICAM1, SBNO2, IL6, CEBPB, CCL2, CD40, LILRB2, CCL20, CCR5, HAMP, SERPINE1, ZC3H12A, SPON2, TNFAIP3, CD14 |
| GO:0030593~neutrophil chemotaxis | 11 | 4.21E-08 | CCL2, C5AR1, S100A8, CCL20, CXCL3, S100A9, ITGA1, FCER1G, IL1B, TREM1, CCL7 |
| GO:0006955~immune response | 24 | 7.45E-08 | IL6, IL1R1, CCL2, CEBPB, C5AR1, IL2RA, AQP9, CXCL3, CCR1, TNFSF14, C1R, CXCL6, CD40, LIF, TNFRSF1A, LILRB2, FCGR2B, CCR5, CCL20, HAMP, IL4R, IL1B, CTSC, THBS1 |
| GO:0030199~collagen fibril organization | 9 | 8.38E-08 | CYP1B1, ADAMTS14, COL3A1, COL1A1, LOX, LOXL2, COL5A1, ANXA2, MMP11 |
| GO:0007229~integrin-mediated signaling pathway | 12 | 2.34E-07 | IBSP, ITGA5, FERMT3, COL3A1, ITGA1, FCER1G, ADAMTS1, ITGA3, ITGB3, ADAM12, ADAM8, ITGB1 |
| GO:0006935~chemotaxis | 13 | 2.55E-07 | PLP2, RNASE2, C5AR1, CCL2, CCR1, CXCL6, CCL7, PLAUR, RAC2, CCL20, CCR5, FOSL1, PLAU |
| GO:0045766~positive regulation of angiogenesis | 11 | 7.90E-06 | CYP1B1, C5AR1, HMOX1, SERPINE1, SPHK1, CHI3L1, IL1B, ZC3H12A, RUNX1, THBS1, ANGPTL4 |
| GO:0042060~wound healing | 9 | 2.34E-05 | DCBLD2, IL6, S100A8, CCL20, COL3A1, LOX, ITGB3, FN1, TIMP1 |
| GO:0019221~cytokine-mediated signaling pathway | 11 | 2.49E-05 | PLP2, TNFRSF1A, IL2RB, IL6, BGN, PODNL1, CCL2, SOCS3, CLCF1, CCR1, IL1B |
| GO:0050731~positive regulation of peptidyl-tyrosine phosphorylation | 9 | 2.81E-05 | LIF, ICAM1, IL6, NRP1, CD44, ITGA5, ITGB3, EHD4, IL11 |
| GO:0032496~response to lipopolysaccharide | 12 | 3.31E-05 | TNFRSF1A, PTGIR, THBD, C5AR1, S100A8, LY96, CXCL3, BDKRB1, CXCL6, CD40, LOXL1, SOD2 |
| GO:0071356~cellular response to tumor necrosis factor | 10 | 3.67E-05 | ICAM1, IL6, CCL2, CCL20, HAMP, CHI3L1, ZC3H12A, COL1A1, THBS1, CCL7 |
| GO:0032755~positive regulation of interleukin-6 production | 7 | 4.91E-05 | LILRB2, IL6, NOD2, MAPK13, FCER1G, IL1B, SPON2 |
| GO:0042742~defense response to bacterium | 11 | 5.94E-05 | TNFRSF1A, NOD2, CEBPB, S100A8, CCL20, HAMP, STAB1, S100A9, BCL3, FCER1G, CXCL6 |
| GO:0007166~cell surface receptor signaling pathway | 15 | 5.96E-05 | IL1R1, IL2RA, CCL2, LY96, CCR1, BDKRB2, BIRC3, SIGLEC9, MARCO, LILRB2, CCR5, CLCF1, TACSTD2, LILRB3, CD14 |
| GO:0071347~cellular response to interleukin-1 | 8 | 8.38E-05 | ICAM1, IL6, CCL2, CEBPB, CCL20, CHI3L1, ZC3H12A, CCL7 |
| GO:0016337~single organismal cell-cell adhesion | 9 | 1.25E-04 | ICAM1, MPZL2, SRPX2, CD44, CD93, COL13A1, FBLIM1, SHC1, ADAM8 |
| GO:0022617~extracellular matrix disassembly | 8 | 1.29E-04 | CD44, MMP19, MMP7, ADAM8, MMP1, MMP11, FN1, TIMP1 |
| GO:0002576~platelet degranulation | 9 | 1.44E-04 | F13A1, SERPINE1, ACTN1, SERPINA1, ITGB3, THBS1, FN1, TIMP1, VCL |
| GO:0042832~defense response to protozoan | 5 | 1.51E-04 | BATF, IL6, IL4R, BCL3, CD40 |
| GO:0006936~muscle contraction | 9 | 1.87E-04 | ACTG2, ACTA2, ITGA1, MYL12A, TPM2, MYOF, TPM4, MYL9, VCL |
| GO:0009612~response to mechanical stimulus | 7 | 2.29E-04 | CCL2, COL3A1, CHI3L1, BDKRB1, BDKRB2, THBS1, FOSL1 |
| GO:0071407~cellular response to organic cyclic compound | 7 | 2.29E-04 | TMEM173, P2RY6, NOD2, CCL2, CYP1B1, CEBPB, IL1B |
| GO:0045087~innate immune response | 18 | 2.32E-04 | S100A8, ELF4, LY96, RELB, S100A9, C1R, C1S, MARCO, NOD2, CD55, TMEM173, C1RL, FCER1G, TREM1, JAK3, PTX3, SPON2, CD14 |
| GO:0006953~acute-phase response | 6 | 2.58E-04 | IL6, CEBPB, HAMP, SERPINA1, FN1, CD163 |
| GO:0070374~positive regulation of ERK1 and ERK2 cascade | 11 | 2.82E-04 | ICAM1, IL6, NOD2, NRP1, CCL2, C5AR1, CD44, CCL20, CCR1, CHI3L1, CCL7 |
| GO:0006968~cellular defense response | 7 | 3.01E-04 | PRF1, LILRB2, C5AR1, CCR5, LY96, FOSL1, ITGB1 |
| GO:0030168~platelet activation | 9 | 3.07E-04 | GNA15, IL6, RAC2, COL3A1, FCER1G, ACTN1, COL1A1, CD40, ITGB3 |
| GO:0070527~platelet aggregation | 6 | 3.28E-04 | FERMT3, ACTN1, MYL12A, ITGB3, MYL9, VCL |
| GO:0007160~cell-matrix adhesion | 8 | 3.71E-04 | CD44, COL13A1, COL3A1, ITGA1, ITGA3, ITGB3, ITGB1, VCL |
| GO:0007267~cell-cell signaling | 13 | 4.08E-04 | NAMPT, LILRB2, PTGIR, NRP1, CCR5, CCL20, STAB1, CCR1, S100A9, IL1B, CXCL6, CCL7, IL11 |
| GO:0030335~positive regulation of cell migration | 11 | 4.20E-04 | COL18A1, ITGA5, FERMT3, CCR1, SPHK1, COL1A1, THBS1, LAMB1, MYADM, CCL7, PLAU |
| GO:0002523~leukocyte migration involved in inflammatory response | 4 | 4.81E-04 | CCL2, S100A8, S100A9, ADAM8 |
| GO:0008284~positive regulation of cell proliferation | 18 | 5.85E-04 | COL18A1, NAMPT, IL6, OSMR, CD248, ESM1, WWTR1, ITGB1, IL11, TIMP1, LIF, RAC2, CLCF1, SHC1, THBS1, FOSL1, RUNX2, FN1 |
| GO:0032760~positive regulation of tumor necrosis factor production | 6 | 6.24E-04 | NOD2, CCL2, LY96, FCER1G, SPON2, CD14 |
| GO:0070098~chemokine-mediated signaling pathway | 7 | 6.27E-04 | CCL2, CCR5, CCL20, CCR1, CXCL3, CXCL6, CCL7 |
| GO:0002693~positive regulation of cellular extravasation | 3 | 6.45E-04 | ICAM1, CCL2, PLVAP |
| GO:2000352~negative regulation of endothelial cell apoptotic process | 5 | 7.19E-04 | ICAM1, SERPINE1, TNFAIP3, IL11, ANGPTL4 |
| GO:0050729~positive regulation of inflammatory response | 7 | 7.28E-04 | TNFRSF1A, CCL2, S100A8, MAPK13, SERPINE1, S100A9, CCL7 |
| GO:0051092~positive regulation of NF-kappaB transcription factor activity | 9 | 8.11E-04 | ICAM1, IL6, NOD2, S100A8, S100A9, SPHK1, IL1B, CD40, ADAM8 |
| GO:0051770~positive regulation of nitric-oxide synthase biosynthetic process | 4 | 8.16E-04 | NAMPT, NOD2, CCL2, CCL20 |
| GO:2000353~positive regulation of endothelial cell apoptotic process | 4 | 0.001027 | COL18A1, CD248, CD40, THBS1 |
| GO:0046718~viral entry into host cell | 7 | 0.001179 | ICAM1, CD55, ITGA5, ANPEP, CTSB, ITGB3, ITGB1 |
| GO:0006508~proteolysis | 18 | 0.001268 | CTSZ, ADAMTS14, MMP19, MMP7, C1R, ANPEP, C1S, MMP1, MMP11, BACE2, C1RL, ADAMTS1, CTSC, CTSB, PRSS23, ADAM8, ADAM12, PLAU |
| GO:0033627~cell adhesion mediated by integrin | 4 | 0.00127 | ICAM1, ITGA5, ITGB3, ITGB1 |
| GO:0050776~regulation of immune response | 10 | 0.00135 | ICAM1, LAIR1, LILRB2, FCGR2B, COL3A1, COL1A1, TREM1, CD40, ITGB1, SIGLEC9 |
| GO:0007596~blood coagulation | 10 | 0.001695 | THBD, F13A1, SH2B3, FCER1G, SERPINA1, COL1A1, ITGB3, PLAU, PLAUR, RAB27A |
| GO:0042127~regulation of cell proliferation | 10 | 0.001761 | SAT1, TNFRSF1A, IL4R, CXCL3, S100A11, NDRG1, CXCL6, CD40, PLAU, TES |
| GO:0031589~cell-substrate adhesion | 4 | 0.001857 | ITGA5, MYO1G, ITGB3, ITGB1 |
| GO:0007249~I-kappaB kinase/NF-kappaB signaling | 6 | 0.001902 | TNFRSF1A, LY96, RELB, BCL3, BIRC3, CD14 |
| GO:0006915~apoptotic process | 19 | 0.001962 | PRF1, IER3, IL2RA, C5AR1, S100A8, S100A9, CHI3L1, TNFSF14, BIRC3, TMEM173, PLK3, TNFAIP8, MAP3K8, ZC3H12A, IL1B, CTSC, TNFAIP3, CD14, PHLDA2 |
| GO:0034605~cellular response to heat | 5 | 0.002091 | HMOX1, ANO1, HSPA6, THBS1, MYOF |
| GO:0008285~negative regulation of cell proliferation | 15 | 0.00235 | B4GALT1, COL18A1, IL6, RARRES1, CYP1B1, ITGA1, S100A11, SOD2, LIF, VDR, IL1B, ADAMTS1, NDRG1, FOSL1, TES |
| GO:0007044~cell-substrate junction assembly | 3 | 0.003134 | ITGA5, ITGB3, FN1 |
| GO:0007568~aging | 9 | 0.003198 | IL6, CCL2, HAMP, COL3A1, BCL2A1, MMP7, CTSC, LOXL2, TIMP1 |
| GO:0043066~negative regulation of apoptotic process | 16 | 0.003244 | IER3, IL2RB, IL6, SOCS3, SPHK1, BCL2A1, BIRC3, TIMP1, PLAUR, SOD2, PLK3, CD44, TNFAIP8, BCL3, THBS1, ANGPTL4 |
| GO:0042730~fibrinolysis | 4 | 0.003477 | SERPINE1, PLAU, ANXA2, PLAUR |
| GO:0043524~negative regulation of neuron apoptotic process | 8 | 0.003477 | NRP1, CCL2, CEBPB, C5AR1, CLCF1, HMOX1, ADAM8, SOD2 |
| GO:0045429~positive regulation of nitric oxide biosynthetic process | 5 | 0.003647 | ICAM1, IL6, IL1B, PTX3, SOD2 |
| GO:0007204~positive regulation of cytosolic calcium ion concentration | 8 | 0.003778 | CD55, GNA15, PTGIR, C5AR1, CCR5, CCR1, BDKRB1, BDKRB2 |
| GO:0007165~signal transduction | 30 | 0.003829 | NAMPT, OSTF1, CCL2, NRP1, S100A9, TNFSF14, CXCL6, GPRC5A, CCL7, TNFRSF1A, VDR, RAC2, CCL20, IL4R, SH2B3, IL1B, SHC1, RUNX1, IL2RB, C5AR1, SPHK1, S100A11, COL15A1, OR51E1, PLAUR, LILRB2, THBD, FCGR2B, PLAU, IGFBP4 |
| GO:0071260~cellular response to mechanical stimulus | 6 | 0.003979 | TNFRSF1A, MMP7, IL1B, TNFSF14, COL1A1, CD40 |
| GO:0034113~heterotypic cell-cell adhesion | 4 | 0.003982 | LILRB2, ITGA5, ITGB3, ITGB1 |
| GO:0002040~sprouting angiogenesis | 4 | 0.005757 | NRP1, ESM1, THBS1, LOXL2 |
| GO:0007159~leukocyte cell-cell adhesion | 4 | 0.005757 | ICAM1, ITGA5, FERMT3, ITGB1 |
| GO:0001503~ossification | 6 | 0.006603 | IBSP, OSTF1, CHRDL2, PDLIM7, RUNX1, RUNX2 |
| GO:0050832~defense response to fungus | 4 | 0.007165 | S100A8, HAMP, S100A9, SPON2 |
| GO:0035987~endodermal cell differentiation | 4 | 0.007165 | ITGA5, COL8A1, LAMB1, FN1 |
| GO:0034097~response to cytokine | 5 | 0.007217 | OSMR, COL3A1, RELB, FOSL1, TIMP1 |
| GO:0050766~positive regulation of phagocytosis | 4 | 0.008759 | FCER1G, IL1B, PTX3, RAB27A |
| GO:0046888~negative regulation of hormone secretion | 3 | 0.009041 | LIF, IL6, IL11 |
| GO:0051918~negative regulation of fibrinolysis | 3 | 0.009041 | THBD, SERPINE1, THBS1 |
| GO:0002675~positive regulation of acute inflammatory response | 3 | 0.009041 | IL6, OSMR, ADAM8 |
| GO:0010803~regulation of tumor necrosis factor-mediated signaling pathway | 4 | 0.009628 | TNFRSF1A, SPHK1, BIRC3, TNFAIP3 |
| GO:0010759~positive regulation of macrophage chemotaxis | 3 | 0.010943 | CCL2, C5AR1, THBS1 |
| GO:0048333~mesodermal cell differentiation | 3 | 0.010943 | ITGA3, ITGB3, ITGB1 |
| GO:0042511~positive regulation of tyrosine phosphorylation of Stat1 protein | 3 | 0.010943 | LIF, TNFRSF1A, CD40 |
| GO:0031663~lipopolysaccharide-mediated signaling pathway | 4 | 0.011511 | CCL2, LY96, IL1B, CD14 |
| GO:0045669~positive regulation of osteoblast differentiation | 5 | 0.011866 | IL6, CEBPB, PDLIM7, FAM20C, RUNX2 |
| GO:0006874~cellular calcium ion homeostasis | 6 | 0.012245 | VDR, CCL2, CCR1, CD40, ATP13A3, CCL7 |
| GO:0043491~protein kinase B signaling | 4 | 0.012526 | PLK3, CCL2, IL1B, CD40 |
| GO:1902042~negative regulation of extrinsic apoptotic signaling pathway via death domain receptors | 4 | 0.012526 | ICAM1, HMOX1, SERPINE1, TNFAIP3 |
| GO:0050777~negative regulation of immune response | 3 | 0.013005 | LILRB2, IL2RA, COL3A1 |
| GO:0043206~extracellular fibril organization | 3 | 0.013005 | LTBP2, COL3A1, COL5A1 |
| GO:0071456~cellular response to hypoxia | 6 | 0.013902 | VASN, ICAM1, HMOX1, LMNA, NDRG1, ADAM8 |
| GO:0050727~regulation of inflammatory response | 5 | 0.014009 | SBNO2, IL1R1, NOD2, BIRC3, TNFAIP3 |
| GO:0002526~acute inflammatory response | 3 | 0.015221 | B4GALT1, IL6, S100A8 |
| GO:0010628~positive regulation of gene expression | 10 | 0.016139 | ACTG2, VDR, IL6, ACTA2, LRRC32, IL1B, ZC3H12A, ITGA3, FN1, RAB27A |
| GO:0007200~phospholipase C-activating G-protein coupled receptor signaling pathway | 5 | 0.016382 | GPR84, P2RY6, HRH1, C5AR1, ANO1 |
| GO:0030194~positive regulation of blood coagulation | 3 | 0.017587 | SERPINE1, S100A9, THBS1 |
| GO:0071354~cellular response to interleukin-6 | 3 | 0.017587 | SBNO2, CCL2, HAMP |
| GO:0008360~regulation of cell shape | 7 | 0.017796 | ICAM1, IL6, CCL2, FBLIM1, MYL12A, CCL7, FN1 |
| GO:0010811~positive regulation of cell-substrate adhesion | 4 | 0.018355 | EGFL6, ITGA5, ITGA3, COL8A1 |
| GO:0034446~substrate adhesion-dependent cell spreading | 4 | 0.018355 | FERMT3, ITGB3, LAMB1, FN1 |
| GO:0042517~positive regulation of tyrosine phosphorylation of Stat3 protein | 4 | 0.018355 | LIF, IL6, SOCS3, CLCF1 |
| GO:0043154~negative regulation of cysteine-type endopeptidase activity involved in apoptotic process | 5 | 0.018993 | IL6, CD44, TNFAIP8, TNFSF14, THBS1 |
| GO:0098609~cell-cell adhesion | 10 | 0.019603 | VASN, SNX9, TACSTD2, S100A11, NDRG1, GPRC5A, VASP, ANXA2, EHD4, TES |
| GO:0051044~positive regulation of membrane protein ectodomain proteolysis | 3 | 0.020097 | SNX9, IL1B, ADAM8 |
| GO:0030330~DNA damage response, signal transduction by p53 class mediator | 3 | 0.020097 | BATF, BCL3, NDRG1 |
| GO:0000187~activation of MAPK activity | 6 | 0.021251 | NOD2, C5AR1, ITGA1, IL1B, SHC1, THBS1 |
| GO:0090026~positive regulation of monocyte chemotaxis | 3 | 0.022748 | CCL2, CCR1, SERPINE1 |
| GO:0030949~positive regulation of vascular endothelial growth factor receptor signaling pathway | 3 | 0.022748 | ITGA5, IL1B, ITGB3 |
| GO:0030334~regulation of cell migration | 5 | 0.023889 | LMNA, ITGB3, PLXND1, PHLDA2, VCL |
| GO:0045599~negative regulation of fat cell differentiation | 4 | 0.02393 | IL6, WWTR1, MMP11, SOD2 |
| GO:0002548~monocyte chemotaxis | 4 | 0.02393 | IL6, CCL2, CCL20, CCL7 |
| GO:0035904~aorta development | 3 | 0.028449 | LOX, PLXND1, LOXL1 |
| GO:0006970~response to osmotic stress | 3 | 0.028449 | PLK3, AQP9, MAPK13 |
| GO:0071363~cellular response to growth factor stimulus | 4 | 0.028646 | IBSP, SHC1, THBS1, EHD4 |
| GO:0018277~protein deamination | 2 | 0.029321 | LOXL2, LOXL1 |
| GO:0035419~activation of MAPK activity involved in innate immune response | 2 | 0.029321 | NOD2, ADAM8 |
| GO:0019442~tryptophan catabolic process to acetyl-CoA | 2 | 0.029321 | KYNU, TDO2 |
| GO:0070488~neutrophil aggregation | 2 | 0.029321 | S100A8, S100A9 |
| GO:0050728~negative regulation of inflammatory response | 5 | 0.029484 | TNFRSF1A, IL2RA, SOCS3, METRNL, TNFAIP3 |
| GO:0051591~response to cAMP | 4 | 0.03032 | THBD, MMP19, COL1A1, FOSL1 |
| GO:0010595~positive regulation of endothelial cell migration | 4 | 0.03032 | NRP1, ZC3H12A, ITGB3, THBS1 |
| GO:0008630~intrinsic apoptotic signaling pathway in response to DNA damage | 4 | 0.032044 | TNFRSF1A, HMOX1, BCL2A1, SOD2 |
| GO:0071230~cellular response to amino acid stimulus | 4 | 0.032044 | COL4A1, CEBPB, COL3A1, COL1A1 |
| GO:0045944~positive regulation of transcription from RNA polymerase II promoter | 23 | 0.033671 | NAMPT, SBNO2, IL6, FOSL2, CEBPB, ELF4, RELB, CD40, WWTR1, IL11, LIF, BATF, TNFRSF1A, VDR, TMEM173, NOD2, SERPINE1, BCL3, ZC3H12A, IL1B, RUNX1, FOSL1, FHOD1 |
| GO:0010951~negative regulation of endopeptidase activity | 6 | 0.033749 | SERPINB8, COL6A3, SERPINE1, SERPINB1, SERPINA1, TIMP1 |
| GO:0009408~response to heat | 4 | 0.033818 | IL6, CCL2, TRPV2, CD14 |
| GO:0046697~decidualization | 3 | 0.034656 | LIF, VDR, CTSB |
| GO:0018149~peptide cross-linking | 4 | 0.037516 | F13A1, COL3A1, THBS1, FN1 |
| GO:0006928~movement of cell or subcellular component | 5 | 0.038518 | ARPC1B, TPM4, ARHGDIB, PLAUR, VCL |
| GO:0042542~response to hydrogen peroxide | 4 | 0.03944 | HMOX1, COL1A1, FOSL1, SOD2 |
| GO:0001934~positive regulation of protein phosphorylation | 6 | 0.040255 | FAM20A, IL1B, CD40, ITGB3, ANXA2, PLAUR |
| GO:0000165~MAPK cascade | 9 | 0.041232 | IL2RB, CCL2, IL2RA, CCR5, MAPK13, IL1B, SHC1, PLVAP, JAK3 |
| GO:0090023~positive regulation of neutrophil chemotaxis | 3 | 0.041334 | C5AR1, RAC2, CXCL3 |
| GO:0043547~positive regulation of GTPase activity | 15 | 0.041518 | ICAM1, SNX9, IL2RB, CCL2, IL2RA, CYTH4, CD40, ITGB1, CCL7, PTGIR, ADAP2, CCL20, SHC1, JAK3, ARHGDIB |
| GO:0001666~response to hypoxia | 7 | 0.042837 | CCL2, HMOX1, THBS1, LOXL2, PLAU, SOD2, ANGPTL4 |
| GO:0016477~cell migration | 7 | 0.042837 | CD248, BDKRB1, JAK3, ITGB3, THBS1, ITGB1, COL5A1 |
| GO:2000391~positive regulation of neutrophil extravasation | 2 | 0.043658 | IL1R1, ADAM8 |
| GO:0002374~cytokine secretion involved in immune response | 2 | 0.043658 | NOD2, TREM1 |
| GO:0070487~monocyte aggregation | 2 | 0.043658 | CD44, IL1B |
| GO:0032602~chemokine production | 2 | 0.043658 | S100A8, S100A9 |
| GO:0044691~tooth eruption | 2 | 0.043658 | FAM20A, COL1A1 |
| GO:0071461~cellular response to redox state | 2 | 0.043658 | VASN, ARHGDIB |
| GO:0045626~negative regulation of T-helper 1 cell differentiation | 2 | 0.043658 | IL4R, JAK3 |
| GO:0048146~positive regulation of fibroblast proliferation | 4 | 0.045504 | FOSL2, SPHK1, ANXA2, FN1 |
| GO:0051602~response to electrical stimulus | 3 | 0.048449 | IL6, CD14, SOD2 |
