# Supplementary material for: BDKRB2 is a novel EMT-related biomarker and predicts poor survival in glioma
Source: Aging (Albany NY). 2021 Mar 3;13(5):7499–516. doi: 10.18632/aging.202614 (PMC7993731; doi:10.18632/aging.202614)
Supplement: Supplementary Table 4 [file aging-13-202614-s005.docx]

**Supplementary Table 4. Gene sets of EMT-related signaling pathways.**

| **Pathway** | **GeneID** |
| --- | --- |
| TGFB | TGFBR1 |
| TGFB | SMAD7 |
| TGFB | TGFB1 |
| TGFB | SMURF2 |
| TGFB | SMURF1 |
| TGFB | BMPR2 |
| TGFB | SKIL |
| TGFB | SKI |
| TGFB | ACVR1 |
| TGFB | PMEPA1 |
| TGFB | NCOR2 |
| TGFB | SERPINE1 |
| TGFB | JUNB |
| TGFB | SMAD1 |
| TGFB | SMAD6 |
| TGFB | PPP1R15A |
| TGFB | TGIF1 |
| TGFB | FURIN |
| TGFB | SMAD3 |
| TGFB | FKBP1A |
| TGFB | MAP3K7 |
| TGFB | BMPR1A |
| TGFB | CTNNB1 |
| TGFB | HIPK2 |
| TGFB | KLF10 |
| TGFB | BMP2 |
| TGFB | ENG |
| TGFB | APC |
| TGFB | PPM1A |
| TGFB | XIAP |
| TGFB | CDH1 |
| TGFB | ID1 |
| TGFB | LEFTY2 |
| TGFB | CDKN1C |
| TGFB | TRIM33 |
| TGFB | RAB31 |
| TGFB | TJP1 |
| TGFB | SLC20A1 |
| TGFB | CDK9 |
| TGFB | ID3 |
| TGFB | NOG |
| TGFB | ARID4B |
| TGFB | IFNGR2 |
| TGFB | ID2 |
| TGFB | PPP1CA |
| TGFB | SPTBN1 |
| TGFB | WWTR1 |
| TGFB | BCAR3 |
| TGFB | THBS1 |
| TGFB | FNTA |
| TGFB | HDAC1 |
| TGFB | UBE2D3 |
| TGFB | LTBP2 |
| TGFB | RHOA |
| WNT | MYC |
| WNT | CTNNB1 |
| WNT | JAG2 |
| WNT | NOTCH1 |
| WNT | DLL1 |
| WNT | AXIN2 |
| WNT | PSEN2 |
| WNT | FZD1 |
| WNT | NOTCH4 |
| WNT | LEF1 |
| WNT | AXIN1 |
| WNT | NKD1 |
| WNT | WNT5B |
| WNT | CUL1 |
| WNT | JAG1 |
| WNT | MAML1 |
| WNT | KAT2A |
| WNT | GNAI1 |
| WNT | WNT6 |
| WNT | PTCH1 |
| WNT | NCOR2 |
| WNT | DKK4 |
| WNT | HDAC2 |
| WNT | DKK1 |
| WNT | TCF7 |
| WNT | WNT1 |
| WNT | NUMB |
| WNT | ADAM17 |
| WNT | DVL2 |
| WNT | PPARD |
| WNT | NCSTN |
| WNT | HDAC5 |
| WNT | CCND2 |
| WNT | FRAT1 |
| WNT | CSNK1E |
| WNT | RBPJ |
| WNT | FZD8 |
| WNT | TP53 |
| WNT | SKP2 |
| WNT | HEY2 |
| WNT | HEY1 |
| WNT | HDAC11 |
| PI3K_AKT | MAPK8 |
| PI3K_AKT | PIK3R3 |
| PI3K_AKT | GRB2 |
| PI3K_AKT | NFKBIB |
| PI3K_AKT | MAP2K6 |
| PI3K_AKT | MAPK9 |
| PI3K_AKT | AKT1 |
| PI3K_AKT | MAPK1 |
| PI3K_AKT | PLCG1 |
| PI3K_AKT | TRIB3 |
| PI3K_AKT | GSK3B |
| PI3K_AKT | MAP2K3 |
| PI3K_AKT | CDKN1A |
| PI3K_AKT | RAC1 |
| PI3K_AKT | RIPK1 |
| PI3K_AKT | AKT1S1 |
| PI3K_AKT | ACTR2 |
| PI3K_AKT | PRKAR2A |
| PI3K_AKT | YWHAB |
| PI3K_AKT | HRAS |
| PI3K_AKT | PDK1 |
| PI3K_AKT | PIKFYVE |
| PI3K_AKT | TBK1 |
| PI3K_AKT | ACTR3 |
| PI3K_AKT | E2F1 |
| PI3K_AKT | MYD88 |
| PI3K_AKT | ITPR2 |
| PI3K_AKT | SQSTM1 |
| PI3K_AKT | RPS6KA1 |
| PI3K_AKT | PTPN11 |
| PI3K_AKT | MAPKAP1 |
| PI3K_AKT | PLCB1 |
| PI3K_AKT | RAF1 |
| PI3K_AKT | CAMK4 |
| PI3K_AKT | RPTOR |
| PI3K_AKT | CFL1 |
| PI3K_AKT | CDK4 |
| PI3K_AKT | TRAF2 |
| PI3K_AKT | GNGT1 |
| PI3K_AKT | UBE2N |
| PI3K_AKT | ADCY2 |
| PI3K_AKT | CDKN1B |
| PI3K_AKT | VAV3 |
| PI3K_AKT | FGF6 |
| PI3K_AKT | ECSIT |
| PI3K_AKT | RALB |
| PI3K_AKT | ARF1 |
| PI3K_AKT | MKNK1 |
| PI3K_AKT | CDK1 |
| PI3K_AKT | PTEN |
| PI3K_AKT | ARHGDIA |
| PI3K_AKT | GRK2 |
| PI3K_AKT | FGF17 |
| PI3K_AKT | DDIT3 |
| PI3K_AKT | AC093012.1 |
| PI3K_AKT | TIAM1 |
| PI3K_AKT | CDK2 |
| PI3K_AKT | SFN |
| PI3K_AKT | PRKCB |
| PI3K_AKT | GNA14 |
| PI3K_AKT | EIF4E |
| PI3K_AKT | CLTC |
| PI3K_AKT | TSC2 |
| PI3K_AKT | FGF22 |
| PI3K_AKT | PPP1CA |
| PI3K_AKT | DUSP3 |
| PI3K_AKT | HSP90B1 |
| PI3K_AKT | IL4 |
| PI3K_AKT | STAT2 |
| PI3K_AKT | SLA |
| PI3K_AKT | EGFR |
| PI3K_AKT | PLA2G12A |
| PI3K_AKT | MAPK10 |
| PI3K_AKT | CALR |
| PI3K_AKT | THEM4 |
| PI3K_AKT | RIT1 |
| PI3K_AKT | MKNK2 |
| PI3K_AKT | PPP2R1B |
| PI3K_AKT | CAB39L |
| PI3K_AKT | ARPC3 |
| PI3K_AKT | PITX2 |
| PI3K_AKT | NCK1 |
| PI3K_AKT | IL2RG |
| PI3K_AKT | PFN1 |
| PI3K_AKT | FASLG |
| PI3K_AKT | NOD1 |
| PI3K_AKT | DAPP1 |
| PI3K_AKT | UBE2D3 |
| PI3K_AKT | CAB39 |
| PI3K_AKT | AP2M1 |
| PI3K_AKT | MAP3K7 |
| PI3K_AKT | PRKAG1 |
| PI3K_AKT | CSNK2B |
| PI3K_AKT | PRKAA2 |
| PI3K_AKT | ATF1 |
| PI3K_AKT | SLC2A1 |
| PI3K_AKT | PIN1 |
| PI3K_AKT | TNFRSF1A |
| PI3K_AKT | LCK |
| PI3K_AKT | RPS6KA3 |
| PI3K_AKT | NGF |
| PI3K_AKT | CXCR4 |
| PI3K_AKT | ACACA |
| PI3K_AKT | SMAD2 |
| PI3K_AKT | PAK4 |
| MAPK | AKT1 |
| MAPK | AKT2 |
| MAPK | AKT3 |
| MAPK | ARRB1 |
| MAPK | ARRB2 |
| MAPK | ATF2 |
| MAPK | ATF4 |
| MAPK | BDNF |
| MAPK | BRAF |
| MAPK | CACNA1A |
| MAPK | CACNA1B |
| MAPK | CACNA1C |
| MAPK | CACNA1D |
| MAPK | CACNA1E |
| MAPK | CACNA1F |
| MAPK | CACNA1G |
| MAPK | CACNA1H |
| MAPK | CACNA1I |
| MAPK | CACNA1S |
| MAPK | CACNA2D1 |
| MAPK | CACNA2D2 |
| MAPK | CACNA2D3 |
| MAPK | CACNA2D4 |
| MAPK | CACNB1 |
| MAPK | CACNB2 |
| MAPK | CACNB3 |
| MAPK | CACNB4 |
| MAPK | CACNG1 |
| MAPK | CACNG2 |
| MAPK | CACNG3 |
| MAPK | CACNG4 |
| MAPK | CACNG5 |
| MAPK | CACNG6 |
| MAPK | CACNG7 |
| MAPK | CACNG8 |
| MAPK | CASP3 |
| MAPK | CD14 |
| MAPK | CDC25B |
| MAPK | CDC42 |
| MAPK | CHP1 |
| MAPK | CHP2 |
| MAPK | CHUK |
| MAPK | CRK |
| MAPK | CRKL |
| MAPK | DAXX |
| MAPK | DDIT3 |
| MAPK | DUSP1 |
| MAPK | DUSP10 |
| MAPK | DUSP14 |
| MAPK | DUSP16 |
| MAPK | DUSP2 |
| MAPK | DUSP3 |
| MAPK | DUSP4 |
| MAPK | DUSP5 |
| MAPK | DUSP6 |
| MAPK | DUSP7 |
| MAPK | DUSP8 |
| MAPK | DUSP9 |
| MAPK | ECSIT |
| MAPK | EGF |
| MAPK | EGFR |
| MAPK | ELK1 |
| MAPK | ELK4 |
| MAPK | FAS |
| MAPK | FASLG |
| MAPK | FGF1 |
| MAPK | FGF10 |
| MAPK | FGF11 |
| MAPK | FGF12 |
| MAPK | FGF13 |
| MAPK | FGF14 |
| MAPK | FGF16 |
| MAPK | FGF17 |
| MAPK | FGF18 |
| MAPK | FGF19 |
| MAPK | FGF2 |
| MAPK | FGF20 |
| MAPK | FGF21 |
| MAPK | FGF22 |
| MAPK | FGF23 |
| MAPK | FGF3 |
| MAPK | FGF4 |
| MAPK | FGF5 |
| MAPK | FGF6 |
| MAPK | FGF7 |
| MAPK | FGF8 |
| MAPK | FGF9 |
| MAPK | FGFR1 |
| MAPK | FGFR2 |
| MAPK | FGFR3 |
| MAPK | FGFR4 |
| MAPK | FLNA |
| MAPK | FLNB |
| MAPK | FLNC |
| MAPK | FOS |
| MAPK | GADD45A |
| MAPK | GADD45B |
| MAPK | GADD45G |
| MAPK | GNA12 |
| MAPK | GNG12 |
| MAPK | GRB2 |
| MAPK | HRAS |
| MAPK | HSPA1A |
| MAPK | HSPA1B |
| MAPK | HSPA1L |
| MAPK | HSPA2 |
| MAPK | HSPA6 |
| MAPK | HSPA8 |
| MAPK | HSPB1 |
| MAPK | IKBKB |
| MAPK | IKBKG |
| MAPK | IL1A |
| MAPK | IL1B |
| MAPK | IL1R1 |
| MAPK | IL1R2 |
| MAPK | JMJD7-PLA2G4B |
| MAPK | JUN |
| MAPK | JUND |
| MAPK | KRAS |
| MAPK | LAMTOR3 |
| MAPK | MAP2K1 |
| MAPK | MAP2K2 |
| MAPK | MAP2K3 |
| MAPK | MAP2K4 |
| MAPK | MAP2K5 |
| MAPK | MAP2K6 |
| MAPK | MAP2K7 |
| MAPK | MAP3K1 |
| MAPK | MAP3K11 |
| MAPK | MAP3K12 |
| MAPK | MAP3K13 |
| MAPK | MAP3K14 |
| MAPK | MAP3K2 |
| MAPK | MAP3K20 |
| MAPK | MAP3K3 |
| MAPK | MAP3K4 |
| MAPK | MAP3K5 |
| MAPK | MAP3K6 |
| MAPK | MAP3K7 |
| MAPK | MAP3K8 |
| MAPK | MAP4K1 |
| MAPK | MAP4K2 |
| MAPK | MAP4K3 |
| MAPK | MAP4K4 |
| MAPK | MAPK1 |
| MAPK | MAPK10 |
| MAPK | MAPK11 |
| MAPK | MAPK12 |
| MAPK | MAPK13 |
| MAPK | MAPK14 |
| MAPK | MAPK3 |
| MAPK | MAPK7 |
| MAPK | MAPK8 |
| MAPK | MAPK8IP1 |
| MAPK | MAPK8IP2 |
| MAPK | MAPK8IP3 |
| MAPK | MAPK9 |
| MAPK | MAPKAPK2 |
| MAPK | MAPKAPK3 |
| MAPK | MAPKAPK5 |
| MAPK | MAPT |
| MAPK | MAX |
| MAPK | MECOM |
| MAPK | MEF2C |
| MAPK | MKNK1 |
| MAPK | MKNK2 |
| MAPK | MOS |
| MAPK | MRAS |
| MAPK | MYC |
| MAPK | NF1 |
| MAPK | NFATC2 |
| MAPK | NFATC4 |
| MAPK | NFKB1 |
| MAPK | NFKB2 |
| MAPK | NGF |
| MAPK | NLK |
| MAPK | NR4A1 |
| MAPK | NRAS |
| MAPK | NTF3 |
| MAPK | NTF4 |
| MAPK | NTRK1 |
| MAPK | NTRK2 |
| MAPK | PAK1 |
| MAPK | PAK2 |
| MAPK | PDGFA |
| MAPK | PDGFB |
| MAPK | PDGFRA |
| MAPK | PDGFRB |
| MAPK | PLA2G10 |
| MAPK | PLA2G12A |
| MAPK | PLA2G12B |
| MAPK | PLA2G1B |
| MAPK | PLA2G2A |
| MAPK | PLA2G2C |
| MAPK | PLA2G2D |
| MAPK | PLA2G2E |
| MAPK | PLA2G2F |
| MAPK | PLA2G3 |
| MAPK | PLA2G4A |
| MAPK | PLA2G4B |
| MAPK | PLA2G4E |
| MAPK | PLA2G5 |
| MAPK | PLA2G6 |
| MAPK | PPM1A |
| MAPK | PPM1B |
| MAPK | PPP3CA |
| MAPK | PPP3CB |
| MAPK | PPP3CC |
| MAPK | PPP3R1 |
| MAPK | PPP3R2 |
| MAPK | PPP5C |
| MAPK | PRKACA |
| MAPK | PRKACB |
| MAPK | PRKACG |
| MAPK | PRKCA |
| MAPK | PRKCB |
| MAPK | PRKCG |
| MAPK | PRKX |
| MAPK | PTPN5 |
| MAPK | PTPN7 |
| MAPK | PTPRR |
| MAPK | RAC1 |
| MAPK | RAC2 |
| MAPK | RAC3 |
| MAPK | RAF1 |
| MAPK | RAP1A |
| MAPK | RAP1B |
| MAPK | RAPGEF2 |
| MAPK | RASA1 |
| MAPK | RASA2 |
| MAPK | RASGRF1 |
| MAPK | RASGRF2 |
| MAPK | RASGRP1 |
| MAPK | RASGRP2 |
| MAPK | RASGRP3 |
| MAPK | RASGRP4 |
| MAPK | RELA |
| MAPK | RELB |
| MAPK | RPS6KA1 |
| MAPK | RPS6KA2 |
| MAPK | RPS6KA3 |
| MAPK | RPS6KA4 |
| MAPK | RPS6KA5 |
| MAPK | RPS6KA6 |
| MAPK | RRAS |
| MAPK | RRAS2 |
| MAPK | SOS1 |
| MAPK | SOS2 |
| MAPK | SRF |
| MAPK | STK3 |
| MAPK | STK4 |
| MAPK | STMN1 |
| MAPK | TAB1 |
| MAPK | TAB2 |
| MAPK | TAOK1 |
| MAPK | TAOK2 |
| MAPK | TAOK3 |
| MAPK | TGFB1 |
| MAPK | TGFB2 |
| MAPK | TGFB3 |
| MAPK | TGFBR1 |
| MAPK | TGFBR2 |
| MAPK | TNF |
| MAPK | TNFRSF1A |
| MAPK | TP53 |
| MAPK | TRAF2 |
| MAPK | TRAF6 |
| Notch | JAG1 |
| Notch | NOTCH3 |
| Notch | NOTCH2 |
| Notch | APH1A |
| Notch | HES1 |
| Notch | CCND1 |
| Notch | FZD1 |
| Notch | PSEN2 |
| Notch | FZD7 |
| Notch | DTX1 |
| Notch | DLL1 |
| Notch | FZD5 |
| Notch | MAML2 |
| Notch | NOTCH1 |
| Notch | PSENEN |
| Notch | WNT5A |
| Notch | CUL1 |
| Notch | WNT2 |
| Notch | DTX4 |
| Notch | SAP30 |
| Notch | PPARD |
| Notch | KAT2A |
| Notch | HEYL |
| Notch | SKP1 |
| Notch | RBX1 |
| Notch | TCF7L2 |
| Notch | ARRB1 |
| Notch | LFNG |
| Notch | PRKCA |
| Notch | DTX2 |
| Notch | ST3GAL6 |
| Notch | FBXW11 |
| Hedgehog | SHH |
| Hedgehog | PTCH1 |
| Hedgehog | NRCAM |
| Hedgehog | NRP1 |
| Hedgehog | SCG2 |
| Hedgehog | AMOT |
| Hedgehog | UNC5C |
| Hedgehog | ADGRG1 |
| Hedgehog | HEY1 |
| Hedgehog | GLI1 |
| Hedgehog | THY1 |
| Hedgehog | SLIT1 |
| Hedgehog | CDK6 |
| Hedgehog | HEY2 |
| Hedgehog | NRP2 |
| Hedgehog | TLE3 |
| Hedgehog | TLE1 |
| Hedgehog | L1CAM |
| Hedgehog | PLG |
| Hedgehog | NKX6-1 |
| Hedgehog | NF1 |
| Hedgehog | RASA1 |
| Hedgehog | ETS2 |
| Hedgehog | RTN1 |
| Hedgehog | CRMP1 |
| Hedgehog | MYH9 |
| Hedgehog | VEGFA |
| Hedgehog | CELSR1 |
| Hedgehog | CNTFR |
| Hedgehog | ACHE |
| Hedgehog | PML |
| Hedgehog | CDK5R1 |
| Hedgehog | VLDLR |
| Hedgehog | OPHN1 |
| Hedgehog | LDB1 |
| Hedgehog | DPYSL2 |
| Hypoxia | PGK1 |
| Hypoxia | PDK1 |
| Hypoxia | GBE1 |
| Hypoxia | PFKL |
| Hypoxia | ALDOA |
| Hypoxia | ENO2 |
| Hypoxia | PGM1 |
| Hypoxia | NDRG1 |
| Hypoxia | HK2 |
| Hypoxia | ALDOC |
| Hypoxia | GPI |
| Hypoxia | MXI1 |
| Hypoxia | SLC2A1 |
| Hypoxia | P4HA1 |
| Hypoxia | ADM |
| Hypoxia | P4HA2 |
| Hypoxia | ENO1 |
| Hypoxia | PFKP |
| Hypoxia | AK4 |
| Hypoxia | FAM162A |
| Hypoxia | PFKFB3 |
| Hypoxia | VEGFA |
| Hypoxia | BNIP3L |
| Hypoxia | TPI1 |
| Hypoxia | ERO1A |
| Hypoxia | KDM3A |
| Hypoxia | CCNG2 |
| Hypoxia | LDHA |
| Hypoxia | GYS1 |
| Hypoxia | GAPDH |
| Hypoxia | BHLHE40 |
| Hypoxia | ANGPTL4 |
| Hypoxia | JUN |
| Hypoxia | SERPINE1 |
| Hypoxia | LOX |
| Hypoxia | GCK |
| Hypoxia | PPFIA4 |
| Hypoxia | MAFF |
| Hypoxia | DDIT4 |
| Hypoxia | SLC2A3 |
| Hypoxia | IGFBP3 |
| Hypoxia | NFIL3 |
| Hypoxia | FOS |
| Hypoxia | RBPJ |
| Hypoxia | HK1 |
| Hypoxia | CITED2 |
| Hypoxia | ISG20 |
| Hypoxia | GALK1 |
| Hypoxia | WSB1 |
| Hypoxia | PYGM |
| Hypoxia | STC1 |
| Hypoxia | ZNF292 |
| Hypoxia | BTG1 |
| Hypoxia | PLIN2 |
| Hypoxia | CSRP2 |
| Hypoxia | VLDLR |
| Hypoxia | JMJD6 |
| Hypoxia | EXT1 |
| Hypoxia | F3 |
| Hypoxia | PDK3 |
| Hypoxia | ANKZF1 |
| Hypoxia | UGP2 |
| Hypoxia | ALDOB |
| Hypoxia | STC2 |
| Hypoxia | ERRFI1 |
| Hypoxia | ENO3 |
| Hypoxia | PNRC1 |
| Hypoxia | HMOX1 |
| Hypoxia | PGF |
| Hypoxia | GAPDHS |
| Hypoxia | CHST2 |
| Hypoxia | TMEM45A |
| Hypoxia | BCAN |
| Hypoxia | ATF3 |
| Hypoxia | CAV1 |
| Hypoxia | AMPD3 |
| Hypoxia | GPC3 |
| Hypoxia | NDST1 |
| Hypoxia | IRS2 |
| Hypoxia | SAP30 |
| Hypoxia | GAA |
| Hypoxia | SDC4 |
| Hypoxia | STBD1 |
| Hypoxia | IER3 |
| Hypoxia | PKLR |
| Hypoxia | IGFBP1 |
| Hypoxia | PLAUR |
| Hypoxia | CAVIN3 |
| Hypoxia | CCN5 |
| Hypoxia | LARGE1 |
| Hypoxia | NOCT |
| Hypoxia | S100A4 |
| Hypoxia | RRAGD |
| Hypoxia | ZFP36 |
| Hypoxia | EGFR |
| Hypoxia | EDN2 |
| Hypoxia | IDS |
| Hypoxia | CDKN1A |
| Hypoxia | RORA |
| Hypoxia | DUSP1 |
| Hypoxia | MIF |
| Hypoxia | PPP1R3C |
| Hypoxia | DPYSL4 |
| Hypoxia | KDELR3 |
| Hypoxia | DTNA |
| Hypoxia | ADORA2B |
| Hypoxia | HS3ST1 |
| Hypoxia | CAVIN1 |
| Hypoxia | NR3C1 |
| Hypoxia | KLF6 |
| Hypoxia | GPC4 |
| Hypoxia | CCN1 |
| Hypoxia | TNFAIP3 |
| Hypoxia | CA12 |
| Hypoxia | HEXA |
| Hypoxia | BGN |
| Hypoxia | PPP1R15A |
| Hypoxia | PGM2 |
| Hypoxia | PIM1 |
| Hypoxia | PRDX5 |
| Hypoxia | NAGK |
| Hypoxia | CDKN1B |
| Hypoxia | BRS3 |
| Hypoxia | TKTL1 |
| Hypoxia | MT1E |
| Hypoxia | ATP7A |
| Hypoxia | MT2A |
| Hypoxia | SDC3 |
| Hypoxia | TIPARP |
| Hypoxia | PKP1 |
| Hypoxia | ANXA2 |
| Hypoxia | PGAM2 |
| Hypoxia | DDIT3 |
| Hypoxia | PRKCA |
| Hypoxia | SLC37A4 |
| Hypoxia | CXCR4 |
| Hypoxia | EFNA3 |
| Hypoxia | CP |
| Hypoxia | KLF7 |
| Hypoxia | CCN2 |
| Hypoxia | CHST3 |
| Hypoxia | TPD52 |
| Hypoxia | LXN |
| Hypoxia | B4GALNT2 |
| Hypoxia | PPARGC1A |
| Hypoxia | BCL2 |
| Hypoxia | GCNT2 |
| Hypoxia | HAS1 |
| Hypoxia | KLHL24 |
| Hypoxia | SCARB1 |
| Hypoxia | SLC25A1 |
| Hypoxia | SDC2 |
| Hypoxia | CASP6 |
| Hypoxia | VHL |
| Hypoxia | FOXO3 |
| Hypoxia | PDGFB |
| Hypoxia | B3GALT6 |
| Hypoxia | SLC2A5 |
| Hypoxia | SRPX |
| Hypoxia | EFNA1 |
| Hypoxia | GLRX |
| Hypoxia | ACKR3 |
| Hypoxia | PAM |
| Hypoxia | TGFBI |
| Hypoxia | DCN |
| Hypoxia | SIAH2 |
| Hypoxia | PLAC8 |
| Hypoxia | FBP1 |
| Hypoxia | TPST2 |
| Hypoxia | PHKG1 |
| Hypoxia | MYH9 |
| Hypoxia | CDKN1C |
| Hypoxia | GRHPR |
| Hypoxia | PCK1 |
| Hypoxia | INHA |
| Hypoxia | HSPA5 |
| Hypoxia | NDST2 |
| Hypoxia | NEDD4L |
| Hypoxia | TPBG |
| Hypoxia | XPNPEP1 |
| Hypoxia | IL6 |
| Hypoxia | SLC6A6 |
| Hypoxia | MAP3K1 |
| Hypoxia | LDHC |
| Hypoxia | AKAP12 |
| Hypoxia | TES |
| Hypoxia | KIF5A |
| Hypoxia | LALBA |
| Hypoxia | COL5A1 |
| Hypoxia | GPC1 |
| Hypoxia | HDLBP |
| Hypoxia | ILVBL |
| Hypoxia | NCAN |
| Hypoxia | TGM2 |
| Hypoxia | ETS1 |
| Hypoxia | HOXB9 |
| Hypoxia | SELENBP1 |
| Hypoxia | FOSL2 |
| Hypoxia | SULT2B1 |
| Hypoxia | TGFB3 |
