# Supplementary material for: BDKRB2 is a novel EMT-related biomarker and predicts poor survival in glioma
Source: Aging (Albany NY). 2021 Mar 3;13(5):7499–516. doi: 10.18632/aging.202614 (PMC7993731; doi:10.18632/aging.202614)
Supplement: Supplementary Table 5 [file aging-13-202614-s006.pdf]

SUPPLEMENTARY TABLE

Supplementary Table 5. Patient characteristics in the TCGA RNA-seq and CGGA_301 microarray data.

Characteristics	TCGA RNA-seq (n=697)	CGGA microarray (n=301)
**Gender**		
male	370	180
female	271	121
NA	56	0
**Age (year)**	47 ± 15	42 ± 12
**Tumor subtype**		
Classical	90	23
Mesenchymal	104	111
Proneural	248	86
Neural	115	81
NA	140	0
**WHO grade**		
Grade II	226	122
Grade III	249	51
Grade IV	167	128
NA	55	0
**Karnofsky Performance Score**	84 ± 14	NA
**IDH mutation status**		
Mut	442	134
WT	245	165
NA	10	2
**1p/19q Codeletion status**		
Codeletion	181	16
Non-codeletion	491	76
NA	25	209
**MGMT promoter status**		
Methylated	461	99
Unmethylated	162	187
NA	74	15

NA: Not Available; KPS: Karnofsky Performance Score; MGMT: O^6-Methylguanine Methyltransferase.
